# Amyotrophic Lateral Sclerosis and Pain: A Narrative Review from Pain Assessment to Therapy

**DOI:** 10.1155/2024/1228194

**Published:** 2024-03-16

**Authors:** Vincenzo Pota, Pasquale Sansone, Sara De Sarno, Caterina Aurilio, Francesco Coppolino, Manlio Barbarisi, Francesco Barbato, Marco Fiore, Gianluigi Cosenza, Maria Beatrice Passavanti, Maria Caterina Pace

**Affiliations:** ^1^Department of Women, Child, General and Specialistic Surgery, University of Campania “L. Vanvitelli”, Naples, Italy; ^2^Multidisciplinary Department of Medical, Surgical and Dental Specialties, University of Campania “L. Vanvitelli”, Naples, Italy; ^3^Santa Maria delle Grazie Hospital, Pozzuoli, Naples, Italy

## Abstract

Amyotrophic lateral sclerosis (ALS) is the most frequent neurodegenerative disease of the motor system that affects upper and lower motor neurons, leading to progressive muscle weakness, spasticity, atrophy, and respiratory failure, with a life expectancy of 2–5 years after symptom onset. In addition to motor symptoms, patients with ALS have a multitude of nonmotor symptoms; in fact, it is currently considered a multisystem disease. The purpose of our narrative review is to evaluate the different types of pain, the correlation between pain and the disease's stages, the pain assessment tools in ALS patients, and the available therapies focusing above all on the benefits of cannabis use. Pain is an underestimated and undertreated symptom that, in the last few years, has received more attention from research because it has a strong impact on the quality of life of these patients. The prevalence of pain is between 15% and 85% of ALS patients, and the studies on the type and intensity of pain are controversial. The absence of pain assessment tools validated in the ALS population and the dissimilar study designs influence the knowledge of ALS pain and consequently the pharmacological therapy. Several studies suggest that ALS is associated with changes in the endocannabinoid system, and the use of cannabis could slow the disease progression due to its neuroprotective action and act on pain, spasticity, cramps, sialorrhea, and depression. Our research has shown high patients' satisfaction with the use of cannabis for the treatment of spasticity and related pain. However, especially due to the ethical problems and the lack of interest of pharmaceutical companies, further studies are needed to ensure the most appropriate care for ALS patients.

## 1. Introduction

Amyotrophic lateral sclerosis (ALS) is a fatal neurodegenerative disorder of the motor system which causes a wide range of debilitating physical, but it has also been associated with extramotor impairments (i.e., mood disorders, cognitive and language impairment, sleep problems, sialorrhea, and pain). ALS is no longer considered a purely motor disease, but a multisystem disease with extramotor involvement [1–3]. Incidence of amyotrophic lateral sclerosis (ALS) may hover around 1.6 cases per 100,000 population, but it has been demonstrated that there is a global variation in prevalence and incidence of ALS, with a higher incidence in some regions of the western Pacific [4]. The pathogenic mechanisms in ALS remain unknown, but several clinical phenotypes have been identified that underlie different molecular and genetic mechanisms. Dominant gene mutations are found in 10% of ALS patients. The mutated genes involved are genes encoding Cu/Zn superoxide dismutase (SOD1), TAR-DNA binding protein 43 (TDP-43), fused in sarcoma/translocated in liposarcoma (FUS/TLS), and C9ORF72. Instead, 90% of patients with unknown familial history are referred as sporadic ALS (sALS), and 5% of these patients are found the same gene mutations seen in familial ALS (fALS) [5].

Several studies have shown that neuronal death in the brain and spinal cord of ALS patients is caused by increased oxidative stress and glutamate excitotoxicity, neuroinflammation, and mitochondrial dysfunction [6–8].

Emerging evidence suggests that the damage not only affects neurons but also neighboring non-neuronal cells such as astrocytes, ependymocytes, and oligodendrocytes, resulting in the loss of these support structures and therefore faster progression of the disease [9, 10].

Another important distinction of patients with ALS is based on anatomical region of neuropathology, resulting in a different pattern of onset and a different prognosis.

Typical or “classical” form of ALS affects simultaneous upper motor neurons (UMNs) as Betz cells in layer V of Brodmann's area 4 and lower motor neurons (LMNs) as alpha motor neurons in the motor nuclei of the brainstem and Rexed lamina IX of the anterior horns in the spinal columns [5].

In patients with the typical form, the first symptoms consist of progressive hypoasthenia and hypotrophy of the limbs, initially asymmetrical and more frequently of the upper limbs. Neurological examination will evaluate both signs of impairment of the peripheral motor neuron (atrophy, weakness, and fasciculations) and of the central motor neuron (hyperexcitable osteotendinous reflexes, hypertonia, and pyramidal signs). The most frequent cause of death in these patients is respiratory failure which occurs on average within 3-5 years of onset.

Atypical form of ALS includes all cases in which there is much longer survival, or pure UMN or LMN involvement [5].

The symptomatology of patients with ALS varies mainly according to the somatic regions primarily affected, therefore depending on whether the onset is spinal or bulbar.

Patients with spinal onset complain of difficulty in making fine voluntary movements of the upper limbs, or they frequently stumble.

Clinical progression follows an anatomical contiguity; in fact, about 85% of patients with spinal onset also manifest bulbar symptoms over time [11].

A quarter of the patients, on the other hand, begin the symptoms with dysphagia or dysatria, and in this case, it is defined as bulbar onset ALS, often associated with cognitive alterations and then followed by involvement of the limbs.

Patients with bulbar onset ALS have the worst prognosis, with the shortest survival (with a median survival of 2 years from the time of diagnosis). The disease has a rapid decline not only motor but also intellectual-cognitive [12].

Regardless of the different forms and onset, the management of patients with ALS is complex and often unsatisfactory. Some aspects of the disease are underestimated and undertreated, first the pain on which we focused in this review.

In the past, ALS was considered a painless condition, but research is assessing that pain is a common symptom in 15% to 85% of patients [13].

The aim of our study is to evaluate the different types of pain, the correlation between pain and disease's stages, the pain assessment tools used for patients with ALS, and the available therapies focusing on the benefits of cannabis.

## 2. Methods

We systematically performed a literature search on databases such as PubMed, Cochrane, Google Scholar, and Embase using these keywords and all the possible combinations, “Nociceptive pain”, “Neuropathic pain”, “Central pain”, “Pain”, “Chronic pain,” “Cannabis”, “Cannabinoids”, “Cannabidiol”, “Amyotrophic Lateral Sclerosis”, “Spasticity”, “Treatments”, “Pharmacotherapeutics”, and “Pain assessment scales”, to identify the therapeutic actions of cannabis in ALS patients with pain. We included 304 articles published from 1964 to 2023 and only if they were clinical studies, clinical trials, RCT, preclinical studies, meta-analysis, systematic reviews, and narrative reviews. We excluded case studies, letters to the editor, grey literature, conference proceedings, case report, and case series. Searches were restricted to studies published in English language.

## 3. Results

### 3.1. Pain in ALS

The new guidelines from the International Association for the Study of Pain (IASP) define pain as “an unpleasant sensory and emotional experience associated with, or resembling that associated with, actual or potential tissue damage.”

In the past, pain was dichotomously divided into nociceptive and neuropathic pain, the former caused by damage to a non-neuronal tissue with activation of nociceptors, and the latter due to an injury or disease of the peripheral somatosensory nervous system.

Recently, the IASP has introduced the term “nociplastic pain” to describe a pain that arises from altered nociception despite no clear evidence of actual or threatened tissue damage causing the activation of peripheral nociceptors or evidence for disease or lesion of the somatosensory system causing the pain [14].

It is important to specify that these three types of pain can be present alone or together in the same pathological state of a patient, and diagnosing the predominant type of pain is important for setting the correct therapy [15].

Among the nonmotor symptoms of ALS, pain is one of the most impacting on the quality of life of these patients and their caregivers.

A positive correlation has been demonstrated between the severity of pain and the level of interference with the quality of life, highlighting an important causal link [16].

The interest of the scientific community has only begun to focus on studying the prevalence of ALS patients' pain in the last two decades. Ganzini et al. have published the first report of the prevalence of pain, suffering, poor quality of life, depression, and hopelessness in people with ALS.

100 patients and 91 caregivers were interviewed, and it was deduced that suffering was rated as 4 or greater on a six-point scale by 20% of subjects with ALS, and 19% rated their pain as 4 or greater on a six-point scale [17].

Nowadays, research has continued in studying the pain of patients with ALS. The most recent analyses estimate a prevalence of pain between 15% [18–20] and 85% of ALS patients [16, 21–26].

Probably, this wide range is due to the different sample size, types of studies conducted, and assessment tools used.

Identifying the type of pain present in ALS patients is fundamental for correct clinical and pharmacological management. Our research has revealed controversial results.

One of the primary causes of pain in ALS is pain with neuropathic features that patients describe as intense and continuous burning, spontaneous tingling, allodynia, or hyperalgesia. These symptoms can affect the distal extremities such as the feet and hands or be widespread [13].

However, several studies have revealed that neuropathic pain is not a component of ALS pain because no patient reached the threshold required to diagnose neuropathic pain [16, 27]; others have demonstrated that neuropathic pain in ALS patients was present in a similar percentage to that found in the general population, from 6.9% to 10.0% [28–31].

Perhaps these prevalence data on neuropathic pain could have undergone an evaluation bias due to the intake of riluzole by ALS patients, because riluzole blocks the presynaptic release of glutamate with possible attenuation of symptoms [28, 32–34].

Other primary causes of pain in ALS patients are cramps and spasticity, which are considered the most reported sources of pain, as evidenced by several studies [23, 35–40].

Caress et al. enrolled forty-one ALS patients in a prospective longitudinal study of the prevalence, frequency, distribution, and severity of muscle cramps. They interviewed patients by telephone from the early stages of the disease, every month, for up to 21 months. At the start of the study, 78% of the participants reported cramps, and of these patients, 34% described the cramp pain as moderate, while 24% as severe. During the study, the percentage of patients recruited with cramps increased to 95%. Moreover, patients with spinal onset had a higher frequency of cramps than patients with bulbar onset [37].

A cross-sectional survey on pain characteristics in amyotrophic lateral sclerosis had already reported very similar data on the incidence of cramps: 63% of 46 enrolled ALS patients [23].

The most involved body sites are the calf and thigh, followed by the hand and foot [23, 37].

It was found that cramps are more frequent during the night and less frequent during the day probably due to movement [23, 41].

The origin of the cramps is explained by the instability of the motor units at the level of the distal motor nerves and by muscle denervation, as shown with needle electromyography studies [42].

Fischer et al. confirmed that motor neuron's death is preceded by severe loss of motor axons, especially larger caliber ones, of the ventral root and significant denervation at the corresponding neuromuscular junctions [43].

Another accredited explanation for cramps and fasciculations was found in the hyperexcitability of motor units after axonal sprouting [23].

Park et al. have shown that anomalies of Na + and K + conductances contribute to the development of membrane hyperexcitability in patients with ALS and the consequent muscle cramps and fasciculations, as well as triggering a neurodegenerative cascade through Ca2 + mediated processes [44].

Another painful symptom is spasticity whose prevalence is between 11% [23] and 36% of patients with ALS [45]. 42.5% of spastic patients reported mild pain, while 16.7% of spastic patients had significant pain with NRS score > 4 [45].

Spasticity is caused by the reduction of suprasegmental control of spinal reflexes and by the alteration of the inhibitory mechanisms present in the spinal cord [46].

Spasticity increases the pathological decline of these patients, causing painful, violent, and paroxysmal contractures with rigidity of the involved muscles [47].

The secondary causes of pain in ALS patients are mainly nociceptive, resulting from non-neural tissue damage with activation of nociceptors due to mechanical or inflammatory stimuli [48].

This type of pain develops with the progression of the disease; in fact, the prolonged immobility associated with atrophy and muscle weakness causes degenerative changes in the musculoskeletal system and the joints, depleted of support [13, 49, 50].

The most frequent affected joints are those of the shoulder and of the hips [21, 49].

Musculoskeletal pain syndromes include adhesive capsulitis (or frozen shoulder) due to inflammation and atrophy of the periscapular muscles [49] and neck pain associated with “head dropping” and low back pain, due to muscle weakness and atrophy, joint degeneration, and unchanged decubitus [29].

Musculoskeletal pain occurs mainly in the later stages of the disease as a result of the stress, exerted on the bones and joints, due to atrophy and damages to the perimysium [39, 51].

Burke et al. evaluated the effectiveness of ultrasound-guided glenohumeral joint injections of local anesthetics and steroids for the management of shoulder pain due to adhesive capsulitis. The procedure offers the advantages of being a rapid outpatient treatment, requiring no ionizing radiation or sedation, and of reducing dependence on systemic pain medications with their potential gastrointestinal, renal, and neurological side effects. Injections into the glenohumeral joint have been shown to be safe and effective, but on their own, they may not completely resolve shoulder pain [52].

Several studies have shown the association between nociceptive pain in ALS patients with reduced mobility and skin pressure, affecting the back, shoulders, neck, and limbs more frequently [17, 18, 21–24, 39].

Patients complain of painful skin lesions such as decubitus ulcers caused by poor mobility [53], but they suffer more due to the ulcers on the face resulting of the mask in noninvasive ventilation (NIV). This is one of the main causes of poor compliance with the NIV [13]. It has recently been shown that neuronal damage and the consequent axonal sprouting with abnormal reorganization of the neuromuscular junction determine a mechanical and electrical dissociation in the muscle with microtrauma and inflammation of the tendons and ligaments as well [16, 54].

Hypotonus and muscle atrophy also contribute to the triggering of the inflammatory response [55].

The constant release of proinflammatory mediators contributes to maintaining a prolonged and intense stimulation of the neurons of the dorsal horn causing neurogenic inflammation and peripheral and central sensitization [56].

Several electrophysiological studies of peripheral nerves in people with ALS have confirmed such generalized sensory system abnormalities [2].

Furthermore, glial cell changes in the spinal cord and brain also underlie the development of chronic pain [57, 58].

Patients with nociceptive pain respond well to nonsteroidal anti-inflammatory drugs (NSAIDs) [59], which do not have effect on neuropathic pain [60].

Lopes et al. found that 46% of the 80 patients enrolled in their study suffered from chronic pain (VAS = 5.18 ± 2.0). Pain of musculoskeletal origin occurred in 40.5% and involved the head/neck area (51%) and the lumbar area (35%). 64.8% of patients took pain medication, and nearly 80% of these were analgesic or NSAIDs, with a relatively good response (70% reported pain relief) [28].

In some patients, pain assessment does not satisfy the diagnostic criteria for either nociceptive or neuropathic pain [47].

Several studies believe that central sensitization could be the explanation [13, 47].

Central sensitization has been found in various pathologies dominated by chronic pain in which there is an increase in the activity of the pathways to facilitate pain and the malfunctioning of the inhibitory pathways descending to pain [15, 61, 62].

Central sensitization is explained by the concept of heterosynaptic enhancement. Heterosynaptic enhancement represents a condition in which sensory inputs, even after being terminated, can amplify the subsequent responses of other unstimulated neurons, resulting in a greater reactivity of central nociceptive neurons to the afferent pathways [62–64].

Our research found that, to date, no studies have addressed the problem of widespread pain in ALS patients through central sensitization analysis, mainly due to the lack of standardized assessment methods. However, neuroimaging studies have shown that anatomical and functional lesions spread beyond the precentral cortices and corticospinal tracts to include the corpus callosum, prefrontal cortex, anterior cingulate cortex, thalamus, insula, amygdala, and midbrain. These functional impairments in ALS patients could explain chronic pain and the cognitive-emotional and affective component of painful sensations [65–67].

Several studies have evaluated the localization of pain in patients with ALS.

Rivera et al. analyzed in a cross-sectional study the characteristics of pain in sixty-four ALS patients (40 males, 24 females: median age 57 years). The most frequent location of pain was in the lower extremities (26%), upper extremities (25%), neck (12%), back (9%), shoulder (7%), hips (6%), abdominal muscles (4%), thorax/ribs (4%), jaw (2%), trapezius (2%), and headache (2%). In most patients, pain was present at more than one location [39].

The majority of the studies in ALS patients have shown similar results on pain localization. The body sites mainly described by patients were lower and upper extremities and the back, but also widespread pain involving multiple body sites [13, 16, 21–24, 28, 68].

Pain intensity has been studied in several studies, with conflicting results.

Chiò et al. conducted a review highlighting that most patients with ALS describe the intensity of pain as mild (NRS < 3) [13, 16, 21–23], but in other studies, pain intensity is mainly recorded as moderate (NRS between 4 and 6) [25] or severe (NRS > 7) [24].

Recently, Hurwitz et al. have published a systematic review and meta-analysis: pain intensity is reported in seven of the included studies by a total of 1426 participants. 78.8% of patients reported moderate pain, 17.5% described severe pain, 1.7% very severe pain, and only 2% indicated mild pain [36].

In 2019, Edge et al. published a study about pain in ALS patients. They recruited 636 patients with ALS. The NRS was completed by 98.3% of patients, and 68.6% of them reported pain; of these, most had mild pain (median score was 2). Precisely, about half reported values of 1–4, and less than 5% reported values of 8–10 [69].

These differences between the results obtained could derive from the different study methods adopted and especially from the transversal nature of the studies analyzed.

### 3.2. Correlation between Pain and Stages of Disease

In literature, we have found several discordant studies about the correlation between pain and disease phases.

Some studies have supposed a prediagnostic role for pain in ALS patients [13].

Patients with ALS described pain as an initial symptom, occurred up to two years before motor impairment [18, 70].

An Italian study showed indirectly similar results, analyzing the intake of analgesic drugs prior to the ALS onset. ALS patients used drugs for pain more frequently than the general population, and this occurred up to 2 years before disease onset [71].

The types of pain most frequently reported at the onset of disease were painful cramps in the hands and legs, muscle spasms [13, 18, 40, 72, 73], and shoulder pain in about 10% of patients [49].

In a cross-sectional study of 108 ALS patients, 55 men and 53 women, the presence of pain at disease onset was retrospectively assessed: 20.4% of patients reported pain at the onset of the disease [40].

Comparable results were found in a cross-sectional study focusing on pain and sensory changes: 80 patients were included, and 21.6% of them described having chronic pain before the development of ALS [28].

Stephens et al. provided a higher percentage in a larger cohort. They recruited ALS patients to complete an online survey on pain. 424 participants responded to the survey, and 75% of the sample reported pain.

Fifty percent of the 318 pain patients reported that the pain preceded the motor symptoms of ALS and was still present at the time of the interview.

Similar to what has been described in other studies [3–10], no correlation was demonstrated between the severity of pain and the disease duration. Pain can be occurred in every stage of the disease, without differences in frequency between the early and late stages [16, 21–24, 30, 39, 74].

In addition to the discordant studies on the presence of pain in the early stages, there is also conflicting evidence on the correlation among the pain intensity and the disease duration and the functional deterioration.

Some studies have suggested no correlation between the pain intensity and disease duration but have shown that pain intensity (PSI) tended to be higher when the ALSFRS-R score was lower [23, 29, 39].

Others have indicated that the pain intensity has strong relationships with loss of function (as measured by ALSFRS-r) and with disease duration [21, 75, 76].

The results of a longitudinal study on the evaluation of the physical and psychological state of 69 patients with ALS found that using the VAS, the intensity of pain increased by 1 point from the first to the last visit. The median number of days between the first and last assessment was 104 (range 35 to 846 days). Between the first and last assessment, increases were evident for pain (2.3 to 3.3; *p* 0.003) and suffering (4.1 to 5.0; *p* 0.03) [77].

Simone et al. evaluated laser-evoked potentials (LEPs) in relation to the clinical characteristics of 24 patients with ALS. This study showed that pain intensity (measured with the VAS) was significantly higher in patients with longer disease duration. Furthermore, the negative correlation between pain intensity and muscle strength suggests that pain may be an indirect symptom of the disease, resulting from motor deficits and posture [78].

Numerous previous studies have showed that pain intensity increases with ongoing disease, evaluating that 20% of examined patients described a change in pain intensity from moderate to severe [17, 79–81].

Furthermore, they reported that pain is more frequent in the final stages of the disease [17, 79].

The literature in favor of the higher frequency of pain in the later stages of ALS is copious [59, 82–86].

These different conclusions that we have found in the literature are mainly due to the dissimilar study designs and different confounding factors. We have noted some limitations in these studies: there are often small patient cohorts and few longitudinal studies and heterogeneity in the assessment techniques used. All this can have repercussions for prompt pharmacological or nonpharmacological intervention.

The need for a standardized approach in assessing all aspects of pain in ALS is becoming increasingly clear.

### 3.3. Pain Assessment Tools

Pain assessment is of fundamental importance to implement the necessary care to obtain the best possible quality of life for the patient.

There are several tools for assessing pain and its characteristics such as intensity, type, location, and interference with QoL. (Supplementary Table 1).

In clinical practice, both one-dimensional (NRS, VAS, VRS, FPS, and WBFPRS) and multidimensional (BPI, MPQ, and PAINAID) scales are used.

One-dimensional scales only measure pain intensity and no other factors, while multidimensional scales also evaluate other aspects of painful sensation.

The numerical evaluation scale (NRS) has the advantage of not requiring any paper support for its use and evaluates the intensity of pain from 0 (no pain) to 10 (the most terrible pain imaginable).

The NRS consists of a numerical scale from 0 to 10 where a value of 0 indicates no pain, while a value of 10 indicates maximum pain.

Values between 1 and 3 indicate the presence of mild pain, and values between 4 and 6 revealed moderate pain, while values between 7 and 10 suggest severe pain.

Its simplicity allows the NRS to be used even in subjects with limited communication skills [30, 69, 87].

Another one-dimensional scale often used to measure pain intensity is the visual analog scale (VAS) [24]. It corresponds to the visual representation of the amplitude of the pain felt by the patient and consists of a predetermined line 10 cm long, where the left extremity corresponds to “no pain,” while the right extremity corresponds to the “worst possible pain.” The patient is asked to draw a sign on the line that represents the level of pain felt.

The scale, as a one-dimensional measure, is used to assess current pain or possibly pain felt in the past 24 hours.

The score is calculated in millimeter, by measuring with a ruler the length of the line between the extreme corresponding to the minimum intensity and the mark placed by the patient. Based on several studies, the following cut-off values have been suggested: from 0 to 4 mm, “no pain”; from 5 to 44 mm, “mild pain”; 45 to 74 mm, “moderate pain”; and 75 to 100 mm, “intense pain.”

The VAS has the advantage of having a high sensitivity but has the disadvantage of requiring a paper support and is difficult to use in patients with visual, physical, or cognitive deficits [24].

Other one-dimensional scales available to clinicians are the Verbal Assessment Scale (VRS), the Faces Pain Scale (FPS), and the Wong-Baker Faces Pain Rating Scale (WBFPRS).

The Verbal Assessment Scale (VRS) associates the level of pain present (no pain, mild, moderate, strong, and unbearable) to a number from 0 to 4. It has a simple use and does not require a paper support but has a low sensitivity.

The Facies Pain Scale (FPS) has several versions. The original version uses the association of six expressive mimic faces, arranged on a horizontal line. The left extremity corresponds to no pain, and the right extremity indicates the worst pain, with a score ranging from 0 to 5. It is easily intuitive but not very sensitive.

The Wong-Baker Faces Pain Rating Scale (WBFPRS) has a similar structure of the six-face FPS, but the score ranges from 0 to 10.

The multidimensional scales instead evaluate pain as a complex sensory experience, and they also analyze the impact of pain on the quality of life of patients, considering different aspects such as the quality of sleep, the ability to carry out normal daily activities, the impact on mood, and the interference of pain at work, on interpersonal relationships.

The Brief Pain Inventory (BPI) is a multidimensional scale structured as a qualitative and quantitative questionnaire that investigates the intensity of pain experienced in the last week and at the time of the interview (with scales numbered 0, “no pain,” to 10, “the most horrible pain imaginable”) and localization of pain. The BPI also provides information on the treatments performed and the relief obtained by the patient, on a scale ranging from 0% (no improvement) to 100% (improvement). Furthermore, BPI evaluates the interference of pain with daily functions, asking the patient to indicate the level of interference on a scale numbered from 0 (no interference) to 10 (total interference). Obviously, this questionnaire is structured for chronic pain in general; therefore, not being specific for ALS, some functions are not considered, such as the ability to walk and interaction with work [16, 21–23, 28, 30, 88, 89].

The McGill Pain Questionnaire (MPQ) is a pain assessment tool developed in 1975 by Melzack and Torgerson. The original version included 102 verbal pain descriptors, which were then reduced to 78 in later versions. The McGill Pain Questionnaire (MPQ) is a multidimensional pain scale used to assess both the quality and intensity of pain self-described by patients [28, 90].

The PAINAD (Pain Assessment IN Advanced Dementia) is a multidimensional scale for pain assessment. It was designed and used for uncooperative patients with significant cognitive impairment.

The final score ranges from 0 (painless) to 10, where the score from 1 to 3 indicates mild pain, the score from 4 to 6 indicates moderate pain, and the score from 7 to 10 indicates severe pain.

We have not found any studies in which this scale has been used to assess pain in ALS patients [91].

In addition to aforementioned pain assessment scales, there are specific questionnaires to assess the possible neuropathic component of pain. These tools are especially useful for the correct pharmacological management of pain in ALS patients.

The first specific instrument developed to measure and analyze pain caused by lesion of the nervous system was the Neuropathic Pain Scale (NPS), developed in 1997 [92, 93].

Later, other scales were developed such as the Leeds Assessment of Neuropathic Symptoms and Signs (LANSS) Pain Scale [94, 95], the Neuropathic Pain Questionnaire (NPQ) [96, 97], the painDETECT (PD-Q) [98], and the Douleur Neuropathique 4 (DN4) [99, 100].

The Neuropathic Pain Scale (NPS) is the first scale designed to qualify and quantify neuropathic pain, and it is also useful for determining the effectiveness of different treatments.

Obviously, the NPS has not been validated in the ALS population; in fact, this represents a limitation in the studies that have used it [39, 92, 93].

The Leeds Assessment of Neuropathic Symptoms and Signs (LANSS) is a scale developed by Bennett that provides immediate clinical information and helps distinguish nociceptive pain from neuropathic pain.

The maximum score that the patient can receive is 24.

A score of less than 12 points makes it unlikely that the patient's symptoms have neuropathic mechanisms, while a score of 12 or higher makes it possible that the neuropathic mechanisms contribute to the pain in the patient. The LANSS scale had a sensitivity of 85%, a specificity of 80%, and a predictive value of 82%.

We have not found in literature any studies on pain in ALS patients that have used this scale [94, 95].

The Neuropathic Pain Questionnaire (NPQ) is another questionnaire designed to differentiate patients with neuropathic pain from patients with no neuropathic pain. Subjects with scores below 0 are predicted to have non-neuropathic pain, while those with scores at or above 0 are predicted to have neuropathic pain. The NPQ can differentiate patients with neuropathic pain from patients with non-neuropathic pain with a sensitivity of 66.6% and a specificity of 74.4% and an accuracy of 71.4%.

The same research group developed the NPQ-Short Form which has 64.5% sensitivity, 78.6% specificity, and 73% accuracy.

We have not found in literature any studies on pain in ALS patients that have used this questionnaire [96, 97].

Freynhagen et al. developed the painDETECT questionnaire (PD-Q), in 2006, during a prospective multicenter study involving 392 patients. The PD-Q proved to be a reliable screening tool with 85% sensitivity and 80% specificity. The PD-Q showed slightly higher sensitivity and specificity than other screening questionnaires for neuropathic pain such as DN4, LANSS, NPQ, or NPS. The painDETECT was initially developed and validated in patients with back pain but has shown applicability to patients with other types of neuropathic pain as well. An important advantage of this questionnaire is that it is easy for the patient to fill in, without first needing any clinical medical examination. Maximum achievable score is 38. A score ≤ 12 indicates that pain is unlikely to have a neuropathic component (probability < 15%), while a score ≥ 19 suggests that pain is likely to have a neuropathic component (probability > 90%). A score between 13 and 18 correlates to an unclear cause for pain [16, 98].

The Douleur Neuropathique 4 (DN4) is a questionnaire used in several studies to evaluate the predominant type of pain in ALS [28–30, 99, 100].

Respondents with a total score4/10 are considered to have neuropathic pain. A cut-off score of 4 has a predictive value of 86%, a sensitivity of 82.9%, and a specificity of 89.9%.

Beswick et al. conducted a systematic review of the assessment of nonmotor symptoms in clinical trials for amyotrophic lateral sclerosis [101].

They evidenced that nonmotor symptoms, including pain, should have been evaluated with ALS-specific assessment tools or validated for use in people with ALS. For these patients, with progressive physical and speech impairment, traditional measures might not be effective in detecting the change in symptoms correctly. Instead, this could be done using validated tools specifically for this type of patients.

The assessments of the prevalence and severity of pain were done using scales and questionnaires that were not specific to ALS patients, which might not be objective enough to detect changes that occur during disease progression or assessment of a pharmacological improvement of symptoms. Using specific or tailored assessment tools for patients with ALS, we could better determine the prevalence and progression of nonmotor symptoms, including pain [47–102].

### 3.4. Pain Treatments in ALS

The insufficient understanding of ALS physiopathology, the few data in the literature, and the absence of guidelines still make difficult to set up a pharmacological therapy in patients with ALS. There is the necessity to establish new therapeutic strategies universally accepted.

The Food and Drug Administration approved two possible disease-modifying therapies that can slow ALS progression, riluzole and edaravone, but they are used in a few countries [103–107].

In Italy, for example, riluzole remained the only drug paid by the National Health System (NHS); instead, edaravone since 2020 is no longer disbursable by the NHS due to a poor proven risk-benefit ratio.

In fact, there is a consensus in the scientific community on the modest efficacy of riluzole in prolonging survival, particularly when taken in the early stages of the disease, while there are still debates regarding the efficacy of edaravone in slowing the progression of the disease [108–111].

Riluzole has a neuroprotective action against glutamatergic excitotoxicity. It inhibits the release of glutamate and was the first drug approved by the FDA for the treatment of ALS in 1995 [108].

A long-term follow-up study evaluated the efficacy of riluzole in a cohort of 415 patients with ALS. Long-term use of riluzole was found to be associated with a better prognosis in patients with ALS, whereas short-term use had little impact on survival [112].

Edaravone scavenges free radicals thereby reducing oxidative stress and thus cell damage. It has been approved for the treatment of ALS in some countries (approved in Japan in 2015, South Korea in 2015, United States in 2017, Canada in 2018, Switzerland in 2019, China in 2019, and Indonesia in 2020) [110, 113].

There are conflicting studies about its ability to modify disease progression [114–116].

The FDA recently approved two new disease-modifying drugs: tofersen [117] and AMX0035 (sodium phenylbutyrate and taurursodiol) [118].

Tofersen is an antisense oligonucleotide for the treatment of ALS patients who have a mutation in the superoxide dismutase 1 (SOD1) gene. The significant decline in neurofilament light chain (NfL) after tofersen treatment confirmed its disease-modifying ability [119], given the proven correlation between the biomarker NfL and ALS progression [120].

AMX0035 is a combination of sodium phenylbutyrate (PB) and taurursodiol designed to reduce neuronal death by mitigating both endoplasmic reticulum (ER) stress and mitochondrial dysfunction. The CENTAUR trial has demonstrated a longer median survival of 6.5 months in patients treated with AMX0035 compared to placebo [121].

Regarding the ALS symptoms like pain, spasticity, cramps, sialorrhea, fatigue, depression, and insomnia, they could be alleviated by pharmacological and nonpharmacological interventions, with a multidisciplinary approach mostly based on good clinical practice [122, 123].

The pharmacological treatments of pain are depending on its different origin.

For neuropathic pain, the use of gabapentin, pregabalin, and tricyclic antidepressants is recommended [50, 124, 125].

The nociceptive pain can be relieved by NSAIDs and paracetamol. In case of joint pain, the intra-articular injections of corticosteroids, alone or combined with lidocaine, are useful [55].

Opioids are second-line drugs for refractory pain. Opioids are used primarily in the advanced stages of ALS to control increased pain associated with insomnia. O'Brien et al. conducted a retrospective analysis of oral morphine use and identified the best tolerated effective dose: a mean 30 (±2.34) mg/24 h oral morphine equivalent, with mean treatment duration of 58 (±18.51) days [126].

An additional one study about oral morphine use, lasted 95 days, suggested that strong opioids could be used safely in palliative care [127].

Despite these analgesic effects, the opioids are associated with notorious side effects like respiratory depression, constipation, antitussive effect, and dependence.

Perhaps, for these side effects, the opioid use is more frequent in a hospice or palliative care unit rather than at home [128, 129].

Furthermore, there are differences between countries in opioid use [129].

Another common source of pain is cramps, which improve with the intake of quinine, benzodiazepine, magnesium, and carbamazepine [23].

Symptomatic treatment of muscle cramps includes sufficient hydration of the patient with correction of any electrolyte imbalances and, if possible, elimination of any causative drugs. A new effective and safe therapeutic option has recently been introduced in patients with ALS: mexiletine, a Na+ channel blocker that reduces persistent sodium currents. A multicenter double-blind placebo-controlled crossover trial of mexiletine 150 mg twice daily was conducted in ALS patients complaining of painful muscle cramps. The frequency and severity of muscle cramps were reduced in 18 of 20 patients, and no serious adverse event occurred [130, 131].

A less safe drug is quinine sulfate because its long-term use might be associated with severe thrombocytopenia, cinchonism, hypoglycemia, hypotension, hearing and visual disturbances, gastrointestinal symptoms, cutaneous effects, conduction abnormalities, arrhythmias, hemolysis, and cardiotoxicity [50, 132, 133].

Due to various adverse events reported, the FDA (Food and Drug Administration) in 2010 launched a risk management plan about its off-label use for leg cramps [134, 135].

A survey on palliative care in the clinical management of ALS patients was conducted among members of the European ALS Study Group. Of the 110 centers consulted, 73 (66%) have completed the questionnaire, getting information from 18 European countries.

For fasciculations and cramps, quinine sulfate is used in 58% of centers, benzodiazepines in 40%, magnesium in 25%, and carbamazepine in 23%.

For spasticity, baclofen is the drug of first choice (93%), followed by tizanidine (38%), dantrolene (36%), and benzodiazepines (36%) [50].

The initial treatment approach for spasticity includes stretching exercises and drugs to reduce pain associated with spasms and stiffness. Muscle relaxants such as baclofen and tizanidine unfortunately have several side effects including aggravation of muscle weakness and the sedative effect [27, 122].

Furthermore, some patients are resistant to treatment or experience side effects that force the reduction of the therapeutic dose of these drugs [136, 137].

In these cases, placement of an intrathecal baclofen pump might be an efficient treatment option [138].

A small study was performed to evaluate the results obtained from the placement of an intrathecal baclofen pump (ITB). Eight patients with intractable spasticity-related pain underwent this treatment, six patients described a pain score reduction, and three of them had a complete resolution of pain. The average reduction in pain scale was 54% after placement of the pump. This result is limited, however, by the small cohort of patients involved and the lack of follow-up which precludes long-term evaluation of the efficacy of intrathecal baclofen for pain [139].

Ashworth et al. [140] systematically reviewed in literature treatments for spasticity in amyotrophic lateral sclerosis, but they identified only one RCT (randomized controlled trial), in which spasticity (as measured by the Ashworth Spasticity Scale) was improved at 3 months following a list of exercises involving most muscle groups of the four limbs and trunk [75].

Precisely, twenty-five ALS patients were randomized to receive a daily exercise program (14 patients) or to perform no physical effort beyond the normal daily activities (11 patients).

Patients were evaluated for one year, every three months. In addition to spasticity, muscle strength, functional status, severity of fatigue, pain, and quality of life were also assessed.

At 3 months, patients who exercised regularly showed less worsening of spasticity and functional status, but not of other parameters. At 6 months, there was no significant difference between groups. At 9 and 12 months, there were too few patients in each group for a statistically significant assessment.

This study has a high risk of bias due to a very small sample size (*N* = 25). It is not possible to conclude whether the exercise program is beneficial, but further research is needed because no additional randomized controlled clinical trials have been conducted on the efficacy of pharmacological and nonpharmacological therapy for the treatment of spasticity in ALS patients [75].

About the nonpharmacological analgesic strategies, the therapeutic ultrasounds, the laser therapy, the transcutaneous electrical nerve stimulation (TENS), and the acupuncture are also mentioned in literature [13, 23, 141, 142].

### 3.5. New Therapeutic Perspectives: Cannabis

In recent years, research has been directed towards new therapeutic strategies, and among these, our review focused on the use of cannabis.

In truth, the use of cannabis for medical purposes has been widespread for thousands of years in different areas of the world [143, 144].

Cannabis is one of the oldest known psychotropic drugs. Its use is estimated as early as around 4000 BC in China to treat emesis, parasitic contaminations, and hemorrhage. Even in India, the properties of cannabis were known, which was used since 1000 BC as an anesthetic and anti-inflammatory [145].

Lately, research has focused on the therapeutic use of cannabis especially for neurological diseases; in fact, cannabis relieves pain and spasticity both in people with multiple sclerosis (MS) than amyotrophic lateral sclerosis (ALS) patients and acts positively on tremor, stiffness, and pain in people with Parkinson's disease (PD) [146, 147]. Cannabinoids are a group of chemical compounds capable of binding to the two receptors of our endocannabinoid system known as CB1 and CB2 [148].

Based on their origins, cannabinoids are classified into three groups.

The first group includes phytocannabinoids or natural cannabinoids such as THC and over 100 other cannabinoid compounds contained in Cannabis sativa [149, 150].

*Δ*9-Tetrahydrocannabinol (THC) is the main psychoactive component present in the female inflorescences of Cannabis sativa (marijuana), and it was isolated in 1964 [151, 152].

Cannabidiol (CBD) is the main nonpsychoactive component of cannabis, and it was isolated in 1940 [153].

The presence of cannabidiol diminishes the psychotropic effects of THC [154, 155].

Other known phytocannabinoids, with minor or no psychoactive properties, are tetrahydrocannabivarin (THCV), cannabinol (CBN), cannabichromene (CBC), cannabicyclol (CBL), and cannabigerol (CBG) [156].

The second group consists of synthetic cannabinoids that bind the CB receptors. These are a diverse set of compounds which include HU-210, Win-55212-2, CP-55,940, JWH-073, JWH-018, and other substances much more potent than THC [157].

Among the synthetic cannabinoids, two important drugs should be mentioned: dronabinol and nabilone, synthetic analogs of Δ9-THC.

Dronabinol and nabilone were both approved by the FDA in 1985 for nausea and emesis in patients undergoing chemotherapy. Dronabinol was approved also for wasting syndrome associated with HIV/AIDS. Several clinical studies have confirmed the benefits of this drug in other pathologies including chronic pain, multiple sclerosis, amyotrophic lateral sclerosis (ALS), fibromyalgia, and dementia [158–160].

The third group of cannabinoids consists of endogenous cannabinoids, derived from arachidonic acid. Anandamide was first identified in 1992 [161] and subsequently 2-arachidonoylglycerol (2-AG) in the CNS [162–164].

The endocannabinoid system is an important endogenous system of intercellular communication involved in many physiological processes, including motor control, pain perception, modulation of the immune system, and neuroprotection.

There are two receptors in this system, CB1 and CB2, both coupled to inhibitory G protein.

CB1 receptor is one of the most common in the central nervous system, especially in the hippocampus, basal ganglia, prefrontal cortex, and cerebellum [165] highlighting the critical role of the endocannabinoid system in motor and cognition function [166].

CB2 receptor is mainly found expressed in immune system's cells but has also been detected in the cerebellum and brainstem [167].

It has been demonstrated that the upregulation of CB2 receptors occurs in the CNS due to injury and inflammatory processes [168, 169], specially on activated microglia, involved in the removal of damaged neurons through mechanisms of phagocytosis and cytotoxicity [170].

Activation of the CB2 receptor gives neuroprotection reducing glial activation and downregulating cytokine and chemokine production [171–176].

Several studies have shown the neuroprotective effects of the endocannabinoid system, such as the reduction of excitotoxicity, oxidative damage, and neuroinflammation through the inhibition of microglia by activating CB1 and especially CB2 receptors [177–181].

The endocannabinoid system (ECS) has multiple connections with the other transmission pathways present in the CNS, suggesting that it might be an interesting therapeutic target especially in neurodegenerative diseases [182–184].

CB1 is expressed in the glutamatergic and GABAergic presynaptic terminals in the brain, spinal cord, and peripheral nerves. The activation of cannabinoid receptors inhibits the release of the excitatory neurotransmitter glutamate and potentiates the effect of the inhibitory neurotransmitter GABA [185–188].

Neurodegenerative diseases show common changes in endocannabinoid levels and CB receptor expression as result of neuroinflammation [189].

Any type of CNS injury or disease is also characterized by increased levels of tumor necrosis factor-alpha (TNF-*α*) which determines the neuronal upregulation of AMPA-type glutamatergic receptors (AMPARs), enhancing significantly glutamatergic excitotoxicity. Therefore, a neuroprotective effect could be achieved by avoiding the increase in TNF-*α* concentration [190–195].

Interestingly, this was obtained with activation of the CB1 receptor. The neuroprotective role of the CB1 receptor was demonstrated by measuring the change in AMPAR receptor expression after exposure to TNF-*α* in the presence or absence of CB1 agonists. CB1 activation blocks TNF-*α*-induced upregulation of AMPAR receptors, thereby protecting neurons from excitotoxic neuroinflammatory death (END) [196].

The pharmacological action on the endocannabinoid system therefore could have an important potential role not only on neuromodulation but also on pain [197–199].

Cannabinoid receptor agonists cause analgesic effects in acute and chronic pain states via spinal and supraspinal pathways, similarly to opioids [200, 201].

Many studies indicate that cannabinoids systemically or topically administered enhance the antinociceptive properties of opioids. Additionally, the antiemetic effect of cannabis can alleviate the nausea associated with opioids [202, 203].

The synergy between *Δ*9-tetrahydrocannabinol and morphine has been demonstrated in many animal models. CB2 receptor agonists produce antinociceptive effects in inflammatory pain models, with activation of the opioid system as well. Furthermore, CB receptor agonists enhance the effect of *μ*-opioid receptor agonists in a variety of animal models, and combinations of cannabinoids and opioids can produce synergistic effects [204, 205].

A double-blind, placebo-controlled study has evaluated the direct effects of opioids combined with cannabis in humans. Cannabis and oxycodone were coadministrated to understand the influence of smoked cannabis on the opioid's antinociceptive effect and to evaluate its interaction. Subtherapeutic doses of oxycodone (2.5 mg) and cannabis, if administered individually, did not cause analgesia in the patients, while if administered together, a significant reduction in pain responses was recorded during the cold pressor test (CPT), thanks to the synergistic action of cannabis and opioids [206].

Moreover, there is evidence of CB2 receptor expression in keratinocytes, which, in presence of CB2 agonists, release endogenous *β*-endorphins to activate opioid receptors in the peripheral nerve endings of sensory neurons [207].

A randomized, double-blind, placebo-controlled crossover study evaluated the analgesic efficacy of vaporized cannabis in patients with pain syndromes caused by nervous system lesions or disease refractory to traditional treatment.

42 patients underwent a standardized inhalation procedure of 4 puffs of vaporized cannabis containing placebo, 2.9% or 6.7% delta-9-tetrahydrocannabinol. This first administration was followed by a second administration at 240 minutes, during which patients inhaled four to eight mouthfuls of cannabis (or placebo). Pain intensity, the primary outcome variable, was rated by patients with a one-dimensional scale ranging from 0 (no pain) to 10 (worst possible pain). It was found that vaporized cannabis had conferred relief from neuropathic pain with decrease in the pain intensity [208].

This is a prospective nonrandomized study of 338 patients who suffered of various chronic pain conditions treated with a decoction of Cannabis Flos 19% for 12 months, in addition to their traditional analgesic drug therapy. Pain intensity, pain disability, anxiety, and depression were recorded at 1, 3, 6, and 12 months. After 12 months of therapy, the data showed a substantial improvement [209].

A recent analysis showed that the legalization of medical cannabis was associated with a reduction in both the prescribing (30%) and dosage of Schedule III opioids in the United States. The data was collected during the period in which the legalization of medical cannabis at the state level was implemented, from 1993 to 2014 [210].

A retrospective mirror-image study enrolled twenty-nine patients who suffered from chronic pain to investigate whether medical cannabis could improve quality of life and pain. After 3 months of treatment, data suggested improvement in QoL, pain reduction, decrease of opioid use, and cost savings [211].

There is also evidence that cannabinoids enhance the analgesic effect of nonsteroidal anti-inflammatory drugs (NSAIDs), allowing the reduction of the NSAIDs required dose and their side effects [212].

Although the pathogenetic mechanisms of ALS are yet unknown, it is believed that an important role in the development and progression of the disease concerns neuroinflammation, oxidative damage, alteration of axonal transport by neurofilaments, and glutamatergic excitotoxicity [6–8, 213].

Furthermore, several studies on animal models have demonstrated the presence of alterations of the endocannabinoid system in symptomatic ALS transgenic mice [214].

CB2 receptors, that have a role in neuroprotection, are upregulated in a mouse model for ALS (G93A-SOD1 mutant mice) [215].

Treatment with the synthetic CB1 and CB2 receptor agonist (WIN55, 212-2) and the synthetic selective CB2 agonist (AM-1241) delays the progression of ALS in animal models [215–221].

Moreover, the neuroprotection of THC is decreased by blockade of the CB1 receptor [222].

A human postmortem study confirmed the data obtained in mouse models: patients with ALS have an overexpression of CB2 receptors on spinal cord microglia following neuronal damage [223, 224].

These data suggest that ALS is associated with changes in the endocannabinoid system and the iatrogenic increase of endocannabinoid tone could slow the disease progression due to the neuroprotective action [179, 225, 226].

In addition, cannabis can relieve the multiplicity of ALS symptoms. The different pharmacological properties, the synergy with various endogenous systems, and the several mechanisms of action highlight that cannabis can manage the heterogeneous symptomatology of ALS patients like pain, spasticity, cramps, dyspnea, sialorrhoea, cachexia, insomnia, anxiety, and depression [227–230].

Approximately 50% of patients with ALS experience significant sialorrhoea due to inability to swallow and to close the mouth and head postural difficulties with the risk of coughing and choking. Excessive saliva causes facial irritation and social embarrassment [231].

Cannabis can cause dry mouth by acting on the CB1 and CB2 receptors present on the salivary glands allowing to avoid the use of anticholinergic drugs which can cause sedation and delirium as side effects [232].

Cannabis can also improve “ALS cachexia” by increasing appetite and reducing catabolism. The CB1 receptor is capable of modulating appetite through presynaptic regulation on orexigenic and anorexigenic neurons [233].

This receptor is abundantly expressed both in areas of the CNS involved in the control of food intake (hypothalamus and brainstem) and in gratification (nucleus accumbens), as well as in the peripheral nervous system, and organs involved in metabolism such as the gastrointestinal tract, liver, and adipose tissue [234, 235].

Moreover, cannabis has a bronchodilator action on the airways as reflected by increases in both PEFR and FEV1, which may be beneficial in ALS patients with respiratory insufficiency [236–240].

Several clinical studies indicate that ALS is associated with high levels of anxiety and depression [241–243].

Cannabis could also act on these symptoms, improving the quality of life of ALS patients [244, 245].

In recent years, academic and industrial efforts have focused on the search for new substances capable of increasing the endocannabinoid tone such as selective inhibitors of the main ECS degradative enzymes, namely, fatty acid amide hydrolase (FAAH) or monoacylglycerol lipase (MAGL) [246–249].

Inhibition of the hydrolyzing enzymes FAAH and MAGL could allow to obtain the desired therapeutic effect without the negative side effects associated with the use of exogenous cannabinoid agonists [250].

Simultaneous inhibition of FAAH and MAGL is known to effectively reduce inflammatory pain by increasing 2-AG and AEA [251] and represents a valid therapeutic strategy to reduce opioid doses in pain treatment [252–254].

Spasticity, a debilitating problem for ALS patients [255], is induced by the lack of inhibition of motor neurons both in the motor cortex and in the spinal cord. Cannabis relieves this symptom by acting mainly on the CB1 receptors located on the synapses of the CNS, with the consequent inhibition of the presynaptic calcium influx and therefore a reduced release of glutamatergic neurotransmitters [256, 257].

Several clinical studies conducted in patients with multiple sclerosis have shown the safety and efficacy of tetrahydrocannabinol (THC) and cannabidiol (CBD); indeed, nabiximols, a combination of THC and CBD, has been approved in Europe for the treatment of spasticity in MS patients since 2010, and it is already used in 15 countries [258–264].

These promising results have encouraged the scientific research to evaluate the use of nabiximols for spasticity in ALS patients too [87, 265].

Riva et al. conducted the first one randomized controlled clinical trial to evaluate the safety and efficacy of nabiximols in ALS patients. They included 59 eligible patients who were asked to complete a diary of their daily levels of spasticity, pain, spasm frequency, and sleep disturbances. Nabiximols was delivered via an oromucosal spray, and each 100 *μ*L actuation delivered 2.7 mg of Δ9-THC and 2.5 mg of cannabidiol. The primary outcome was the change in the score on modified Ashworth Scale (MAS) scores in the active group compared with the placebo group.

After 6 weeks, the MAS score significantly improved in the nabiximols group compared to the control group. Additionally, there was a significant reduction in pain, but no significant differences were noted between groups for sleep quality, muscle strength, and scores on ALSFS-R. Nabiximols was overall well tolerated. No participants permanently discontinued treatment during the double-blind phase of the study, and no serious adverse events occurred in either group [265].

An observational study was conducted as a retrospective, single-center, cross-sectional cohort study with the aim of assessing the level of satisfaction with the use of an oromucosal spray containing THC:CBD for the symptomatic management of spasticity in 32 ALS patients [87].

Each 100 *μ*L actuation contained 2.7 mg of THC and 2.5 mg of CBD. The patient's perception of spasticity and associated pain and cramps was recorded with the one-dimensional NRS scale [266, 267].

The NPS has also been used to examine patients' attitudes towards their THC:CBD treatment. Patients were asked the question, “How likely is it that you would recommend THC:CBD to a friend or colleague who suffers from ALS and spasticity?” The answers were evaluated on a numerical scale ranging from 0 (absolutely improbable recommendation) to 10 (highest probability of recommendation).

THC:CBD treatment satisfaction was assessed using the TSQM-9 (Medication Treatment Satisfaction Questionnaire), a validated rating scale containing nine questions [268].

Most patients rated spasticity as severe (24.2%, *n* = 8) or moderate (48.5%, *n* = 16). Of the ALS patients surveyed, 70% (*n* = 23) reported pain.

The overall NPS score was +4.9 points, which translates into a moderately positive recommend rate. In particular, patients with moderate to severe spasticity (NRS ≥ 4) were highly likely to recommend (NPS: +28) treatment with THC:CBD, unlike patients with mild spasticity (NRS < 4) which were unwilling to recommend THC:CBD to other patients (NPS: -44). Another interesting data is the correlation found between the severity of the symptoms and the dose of THC:CBD applied. A mean number of 7.3 (±6.0) actuations was used in patients with severe spasticity, while 3.5 (±2.2) actuations per day were used in patients with mild spasticity.

Evaluation of TSQM-9 showed a high general level of satisfaction with THC:CBD treatment in the majority of ALS patients studied (84% of patients).

Unfortunately, this study evaluated a small sample of patients (*n* = 32), and 40% of them (*n* = 16) discontinued THC:CBD treatment during the study [87].

Cannabis use has also been evaluated for cramps in ALS patients. In fact, the distribution of CB1 receptors in the presynaptic terminals in the brain, spinal cord, and peripheral nerves suggests that its use could be more advantageous than drugs that act only centrally (gabapentin) or only peripherally (quinine sulfate) [269, 270].

Weber et al. conducted a randomized, double-blind, placebo-controlled crossover study of 22 ALS patients who suffered from moderate or severe daily cramps. Adult patients with a mean daily cramp severity score of 4 or greater were eligible. The patients were randomly assigned to receive 5 mg THC twice daily followed by placebo or vice versa. Each treatment period lasted 2 weeks and was preceded by a 2-week drug-free observation period. The primary outcome was the change in cramp intensity assessed with the VAS. Secondary outcome measures included the number of cramps per day, the intensity of fasciculations (VAS) as well as the quality of life (ALSAQ-40), the quality of sleep (SDQ), the appetite (FAACT), and the depression (HADS). THC was well tolerated, but there was no evidence for a treatment effect on cramp intensity, cramp frequency, fasciculation intensity, or any of the other secondary outcome measures. This may be, in part, due to the limited 2-week treatment period and poor knowledge of the pharmacokinetics of THC in ALS patients so with consequent difficulty to determine the correct length of the wash period. Another confounding factor is the natural course of cramps, which is unknown [271].

Amtman et al. [228] conducted a survey with 131 ALS patients. This study is the first anonymous survey of ALS patients regarding cannabis use, published in 2004, well before the official legalization of cannabis in the United States. Only 10% of 131 patients (*n* = 13) reported having used cannabis in the last 12 months, and all of them had already used cannabis in their own life before to have the ALS diagnosis.

Respondents were asked to rate the relief obtained from cannabis use for each symptom on a scale numbered from 0 (not at all) to 4 (completely relieves the symptom). Respondents reported that cannabis use helped moderately with depression, loss of appetite, spasticity, sialorrhoea, and pain.

Duration of symptom relief was also rated using a scale from 0 (no relief) to 6 (more than nine hours). Relief for depression was the longest lasting (averaging two to three hours), while other symptoms were relieved for an average of about an hour or less.

Unfortunately, the survey has several limitations: the 13 cannabis users may not represent the true percentage of ALS patients who use cannabis. Furthermore, 75% of respondents were male and 25% female; therefore, the female percentage is slightly lower than the real one estimated in the general ALS population because men develop ALS at 1.3-1.56 times the rate of women [272, 273].

In 2023, Lacroix et al. published a study on the use of therapeutic cannabis in ALS patients in France. The study analyzed online questionnaires compiled by ALS patients about their “real-life” situation to better understand patients' opinions on medical cannabis. There were 129 respondents and 28 reported using cannabis (21.7%) to relieve ALS symptoms. All cannabis users were men. Regarding frequency of use, 12 participants reported daily use (42.9%), seven weekly use (25%), and seven occasional use (25%). Oral or sublingual administration was the most reported route (*n* = 19, 67.9%), followed by smoking (*n* = 5, 17.9%) and vaping (*n* = 2, 7.1%). One participant reported applying a skin cream. The concentration of the cannabinoids was not well known by users. Regarding the different purchasing methods, 13 participants reported having purchased products in shops (46.4%), 11 on the Internet (39.3%), six in the street (dealer) (21.4%), two self-cultivation (7.1%), one in another European country, and one in a hospital pharmacy because he was a participant in the ANSM experimentation campaign. The majority of patients declared benefits on both motor symptoms (stiffness, cramps, and fasciculations) and nonmotor symptoms (sleep quality, pain, emotional state, quality of life, and depression). Only eight patients reported minor adverse reactions like drowsiness, euphoria, and dry mouth. Furthermore, 15 participants reported an overall positive impression of the change (53.6%). From this study, it emerges that cannabinoids could be an important treatment options for the symptoms of ALS, but at the same time, there is the need to conduct studies on a larger number of patients, to have formulation pharmaceuticals and precise dosages, and the possibility of accessing cannabis through safer routes [274].

The bioavailability and pharmacokinetics of cannabis change depending on the chosen mode of administration. Besides the concentration of THC and other phytocannabinoids in marijuana varies considerably depending on the genetic characteristics of the plant, on the different methods of cultivation and processing [275].

The most common and traditional use of cannabis is through rolled cigarettes or pipes, used to smoke dried cannabis flowers [276].

The main advantage of smoking is the rapid onset of effect and easy dose titration. Cannabinoids present in inhaled smoke are rapidly absorbed and easily cross the blood-brain barrier due to their fat solubility. Obviously, this route of administration carries risks for the respiratory system [277].

To date, healthier formulations are available: vaporized, ingested orally, applied topically, or administered via other routes.

Oral cannabis products are available as both foods and beverages with varying percentages of THC and CBD [278–280].

In addition, cannabis oils and tinctures are also edibles. This mode of administration is preferred by women, the elderly, and patients who use cannabis for medicinal purposes [281–283].

The vaporization of cannabinoids is possible as they are volatile compounds that vaporize at a temperature much lower than combustion, exposing cannabis users to fewer toxicants as carbon monoxide [284, 285].

Heated air is drawn in, and the vaporized active compounds are inhaled and rapidly absorbed [286].

Cannabis vaporizers can heat dried buds or aerosolize the cannabinoids/terpenes for inhalation. Vaporizers represent the ideal choice for patients using medical cannabis as they pose a lower risk to health by not creating combustion products [282, 283, 287].

Patients describe better taste, stronger effects, and greater confidentiality in use (for example, reduced odor) compared to smoked cannabis [288, 289].

The data demonstrate that vaping results in fewer respiratory symptoms than conventionally smoked cannabis, but the long-term effects are still unclear [290].

Another available route of administration is the transdermal one. Topical products can be THC or CBD-dominant, with several possible formulations as lotions, gels, creams, ointments, and patches. Finally, new products have been introduced such as sublingual sprays which have the advantage of bypassing liver metabolism and rectal and vaginal suppositories [276, 279].

Medical cannabis has great therapeutic potential, but its use in clinical practice presents several problems.

First, pharmaceutical companies have little incentive to support large clinical trials because medical cannabis is not protected by any patent [291].

Obviously, the lack of clinical data discourages many clinicians from prescribing it, opting for other drugs. The forms of cannabis that have been approved for medical use are very expensive, and sometimes the pills are difficult for patients with dysphagia and nausea to consume. It has been argued that smoked cannabis is more effective as it crosses the blood-brain barrier quickly. Unfortunately, with this method of administration, it is difficult to determine the effective concentration of the active component [292, 293].

Scientific literature on appropriate dosing regimens for the medicinal use of cannabis is lacking. Carter et al. conducted a study highlighting the need for guidelines on the rational dosing of cannabis. They used the FDA-accepted guidelines on dronabinol prescribing as the basis for making natural cannabis dosing recommendations. The prescribed dose of dronabinol for appetite stimulation is 2.5 mg twice daily, to be taken before lunch and dinner. For nausea, vomiting, and pain, the dosage is 5 mg/m^2^. If the 5 mg dose is ineffective, incremental increases of 2.5 mg are recommended, up to a maximum of 15 mg. The same dose can be taken every 2-4 hours for a maximum of 4-6 doses per day. Regardless of the clinical problem, the maximum total recommended dose of dronabinol is 15 mg/m^2^ four to six times per day or approximately 100 to 120 mg/day. Applying the known pharmacokinetics of cannabis, compared to a conservative dronabinol dosing model of 2.5 to 60 mg/day, they calculated doses for cannabis containing particular percentages of THC [294].

In addition to the problems mentioned above, often the medical use of cannabis is avoided due to the skepticism that can interest both uninformed patients and doctors. Indeed, a common obstacle to the use of cannabis is the fear of adverse effects on the nervous system, cardiorespiratory system, and mental health [295].

Long-term cannabis use is most closely related to risk of addiction [296, 297], dose-related cognitive changes, and possible long-term morpho functional brain alterations. Obviously, as happens with any drug, a conscious and accurate assessment of the risk-benefit ratio is necessary [298–300].

Furthermore, government restrictions and different laws in force between states can hinder its use [301].

A large survey of cannabis use in patients with ALS found that the main reason ALS patients did not use it was their inability to obtain it, whether for legal or financial reasons or lack of safe access [228].

In this review, we have examined the various therapeutic properties of cannabis to encourage its use where conventional therapies are not applicable.

Standardized and safe protocols are needed, which can only be obtained by increasing scientific research [302].

## 4. Limitations

There are several limitations in our review. Many aspects of the ALS pathophysiology and associated pain are not yet fully understood to enable therapeutic progresses. Furthermore, the different types of conducted studies may have created important evaluation biases. A longitudinal approach is needed in these patients to better understand the impact of ALS and pain from the earliest stages. Another major limitation in the progress of research in this medical-scientific field is the impact of the frailty of ALS patients on the ethically correct design of RCTs with placebo. Obviously, the nature of this pathology and the painful complex associated pain symptomatology prevents these fragile patients from being subjected to the use of placebos or from denying multidisciplinary assistance.

## 5. Conclusion

Patients with ALS can experience pain of various etiologies. Based on the available published works, pain should be considered as a symptom to be valued from the early stages of the disease. Fundamental is the development of standardized and specific pain assessment tools for patients with ALS. The heterogeneity of the pathophysiological mechanisms of pain requires at the same time a personalized and multidisciplinary clinical approach. Pain management also has positive psychological effects on ALS patients, thereby influencing the patient's and caregiver's quality of life. However, specific guidelines on pain management in ALS patients are not available, and the different potential pain treatments have not been subjected to randomized trials. Only full knowledge of this disease might allow to use the right pharmacological and nonpharmacological approaches, with the creation of guidelines uniformly accepted by the scientific community.

## Supplementary Materials

Supplementary Materials Table 1: pain assessment tool.

## References

[1] Hammad M., Silva A., Glass J., Sladky J. T., Benatar M. (2007). Clinical, electrophysiologic, and pathologic evidence for sensory abnormalities in ALS. *Neurology*.

[2] Pugdahl K., Fuglsang-Frederiksen A., de Carvalho M. (2007). Generalised sensory system abnormalities in amyotrophic lateral sclerosis: a European multicentre study. *Journal of Neurology, Neurosurgery, and Psychiatry*.

[3] Isak B., Tankisi H., Johnsen B. (2016). Involvement of distal sensory nerves in amyotrophic lateral sclerosis. *Muscle & Nerve*.

[4] Xu L., Liu T., Liu L. (2020). Global variation in prevalence and incidence of amyotrophic lateral sclerosis: a systematic review and meta-analysis. *Journal of Neurology*.

[5] Grad L. I., Rouleau G. A., Ravits J., Cashman N. R. (2017). Clinical spectrum of amyotrophic lateral sclerosis (ALS). *Cold Spring Harbor Perspectives in Medicine*.

[6] Miller R. G., Jackson C. E., Kasarskis E. J. (2009). Practice Parameter update: The care of the patient with amyotrophic lateral sclerosis: Multidisciplinary care, symptom management, and cognitive/behavioral impairment (an evidence-based review) Report of the Quality Standards Subcommittee of the American Academy of Neurology. *Neurology*.

[7] Gurney M. E., Pu H., Chiu A. Y. (1994). Motor neuron degeneration in mice that express a human Cu, Zn superoxide dismutase mutation. *Science*.

[8] Weiss M. D., Ravits J. M., Schuman N., Carter G. T. (2006). A4V superoxide dismutase mutation in apparently sporadic ALS resembling neuralgic amyotrophy. *Amyotrophic Lateral Sclerosis*.

[9] Suzuki M., Klein S., Wetzel E. A. (2010). Acute glial activation by stab injuries does not lead to overt damage or motor neuron degeneration in the G93A mutant SOD1 rat model of amyotrophic lateral sclerosis. *Experimental Neurology*.

[10] Boillée S., Vande Velde C., Cleveland D. W. W. (2006). ALS: a disease of motor neurons and their nonneuronal neighbors. *Neuron*.

[11] Armon C., Moses D. (1998). Linear estimates of rates of disease progression as predictors of survival in patients with ALS entering clinical trials. *Journal of the Neurological Sciences*.

[12] Mitsumoto H., Del Bene M. (2000). Improving the quality of life for people with ALS: the challenge ahead. *Amyotrophic Lateral Sclerosis and Other Motor Neuron Disorders*.

[13] Chiò A., Mora G., Lauria G. (2017). Pain in amyotrophic lateral sclerosis. *Lancet Neurology*.

[14] Raja S. N., Carr D. B., Cohen M. (2020). The revised International Association for the Study of Pain definition of pain: concepts, challenges, and compromises. *Pain*.

[15] Nijs J., Apeldoorn A., Hallegraeff H. (2015). Low back pain: guidelines for the clinical classification of predominant neuropathic, nociceptive, or central sensitization pain. *Pain Physician*.

[16] Wallace V. C., Ellis C. M., Burman R., Knights C., Shaw C. E., Al-Chalabi A. (2014). The evaluation of pain in amyotrophic lateral sclerosis: a case controlled observational study. *Amyotroph Lateral Scler Frontotemporal Degener*.

[17] Ganzini L., Johnston W. S., Hoffman W. F. (1999). Correlates of suffering in amyotrophic lateral sclerosis. *Neurology*.

[18] de Castro-Costa C. M., Oriá R. B., Machado-Filho J. A. (1999). Amyotrophic lateral sclerosis. Clinical analysis of 78 cases from Fortaleza (northeastern Brazil). *Arquivos de Neuro-Psiquiatria*.

[19] Truini A., Biasiotta A., Onesti E. (2015). Small-fibre neuropathy related to bulbar and spinal-onset in patients with ALS. *Journal of Neurology*.

[20] Dalla Bella E., Lombardi R., Porretta-Serapiglia C. (2016). Amyotrophic lateral sclerosis causes small fiber pathology. *European Journal of Neurology*.

[21] Chiò A., Canosa A., Gallo S. (2012). Pain in amyotrophic lateral sclerosis: a population-based controlled study. *European Journal of Neurology*.

[22] Stephens H. E., Lehman E., Raheja D. (2016). Pain in amyotrophic lateral sclerosis: patient and physician perspectives and practices. *Amyotroph Lateral Scler Frontotemporal Degener*.

[23] Hanisch F., Skudlarek A., Berndt J., Kornhuber M. E. (2015). Characteristics of pain in amyotrophic lateral sclerosis. *Brain and Behavior*.

[24] Pizzimenti A., Aragona M., Onesti E., Inghilleri M. (2013). Depression, pain and quality of life in patients with amyotrophic lateral sclerosis: a cross-sectional study. *Functional Neurology*.

[25] Jensen M. P., Abresch R. T., Carter G. T., McDonald C. M. (2005). Chronic pain in persons with neuromuscular disease. *Archives of Physical Medicine and Rehabilitation*.

[26] Abe Y., Miyashita M., Ito N. (2008). Attitude of outpatients with neuromuscular diseases in Japan to pain and use of analgesics. *Journal of the Neurological Sciences*.

[27] EFNS Task Force on Diagnosis and Management of Amyotrophic Lateral Sclerosis, Andersen P. M., Abrahams S. (2012). EFNS guidelines on the clinical management of amyotrophic lateral sclerosis (MALS)--revised report of an EFNS task force. *European Journal of Neurology*.

[28] Lopes L. C. G., Galhardoni R., Silva V. (2018). Beyond weakness: characterization of pain, sensory profile and conditioned pain modulation in patients with motor neuron disease: a controlled study. *European Journal of Pain*.

[29] Moisset X., Cornut-Chauvinc C., Clavelou P., Pereira B., Dallel R., Guy N. (2016). Is there pain with neuropathic characteristics in patients with amyotrophic lateral sclerosis? A cross-sectional study. *Palliative Medicine*.

[30] An R., Li Y., He X. (2021). The evaluation of pain with nociceptive and neuropathic characteristics from three different perspectives in amyotrophic lateral sclerosis patients: a case controlled observational study in Southwestern China. *Neural Plasticity*.

[31] van Hecke O., Austin S. K., Khan R. A., Smith B. H., Torrance N. (2014). Neuropathic pain in the general population: a systematic review of epidemiological studies. *Pain*.

[32] Miller R. G., Mitchell J. D., Lyon M., Moore D. H. (2007). Riluzole for amyotrophic lateral sclerosis (ALS)/motor neuron disease (MND). *Cochrane Database of Systematic Reviews*.

[33] Moon E. S., Karadimas S. K., Yu W. R., Austin J. W., Fehlings M. G. (2014). Riluzole attenuates neuropathic pain and enhances functional recovery in a rodent model of cervical spondylotic myelopathy. *Neurobiology of Disease*.

[34] Poupon L., Lamoine S., Pereira V. (2018). Targeting the TREK-1 potassium channel via riluzole to eliminate the neuropathic and depressive-like effects of oxaliplatin. *Neuropharmacology*.

[35] Swash M., Czesnik D., de Carvalho M. (2019). Muscular cramp: causes and management. *European Journal of Neurology*.

[36] Hurwitz N., Radakovic R., Boyce E., Peryer G. (2021). Prevalence of pain in amyotrophic lateral sclerosis: a systematic review and meta-analysis. *Amyotroph Lateral Scler Frontotemporal Degener.*.

[37] Caress J. B., Ciarlone S. L., Sullivan E. A., Griffin L. P., Cartwright M. S. (2016). Natural history of muscle cramps in amyotrophic lateral sclerosis. *Muscle Nerve*.

[38] Stephens H. E., Joyce N. C., Oskarsson B. (2017). National study of muscle cramps in ALS in the USA. *Amyotroph Lateral Scler Frontotemporal Degener*.

[39] Rivera I., Ajroud-Driss S., Casey P. (2013). Prevalence and characteristics of pain in early and late stages of ALS. *Amyotroph Lateral Scler Frontotemporal Degener*.

[40] Taga A., Schito P., Trapasso M. C., Zinno L., Pavesi G. (2019). Pain at the onset of amyotrophic lateral sclerosis: a cross-sectional study. *Clinical Neurology and Neurosurgery*.

[41] Forshew D. A., Bromberg M. B. (2003). A survey of clinicians' practice in the symptomatic treatment of ALS. *Amyotrophic Lateral Sclerosis and Other Motor Neuron Disorders*.

[42] Miller T. M., Layzer R. B. (2005). Muscle cramps. *Muscle & Nerve*.

[43] Fischer L. R., Culver D. G., Tennant P. (2004). Amyotrophic lateral sclerosis is a distal axonopathy: evidence in mice and man. *Experimental Neurology*.

[44] Park S. B., Kiernan M. C., Vucic S. (2017). Axonal excitability in amyotrophic lateral sclerosis: axonal excitability in ALS. *Neurotherapeutics*.

[45] Verschueren A., Grapperon A. M., Delmont E., Attarian S. (2021). Prevalence of spasticity and spasticity-related pain among patients with amyotrophic lateral sclerosis. *Revue Neurologique (Paris)*.

[46] Mayer N. H. (1997). Clinicophysiologic concepts of spasticity and motor dysfunction in adults with an upper motoneuron lesion. *Muscle & Nerve: Official Journal of the American Association of Electrodiagnostic Medicine*.

[47] Delpont B., Beauvais K., Jacquin-Piques A. (2019). Clinical features of pain in amyotrophic lateral sclerosis: a clinical challenge. *Revue Neurologique (Paris)*.

[48] Smart K. M., Blake C., Staines A., Doody C. (2010). Clinical indicators of 'nociceptive', 'peripheral neuropathic' and 'central' mechanisms of musculoskeletal pain. A Delphi survey of expert clinicians. *Manual Therapy*.

[49] Ho D. T., Ruthazer R., Russell J. A. (2011). Shoulder pain in amyotrophic lateral sclerosis. *Journal of Clinical Neuromuscular Disease*.

[50] Borasio G. D., Shaw P. J., Hardiman O. (2001). Standards of palliative care for patients with amyotrophic lateral sclerosis: results of a European survey. *Amyotrophic Lateral Sclerosis and Other Motor Neuron Disorders*.

[51] Borasio G. D., Voltz R., Miller R. G. (2001). Palliative care in amyotrophic lateral sclerosis. *Neurologic Clinics*.

[52] Burke K. M., Ellrodt A. S., Joslin B. C. (2023). Ultrasound-guided glenohumeral joint injections for shoulder pain in ALS: a case series. *Frontiers in Neurology*.

[53] Hayashi T., Narita Y., Okugawa N., Hamaguchi E., Shibahara M., Kuzuhara S. (2007). Pressure ulcers in ALS patients on admission at a university hospital in Japan. *Amyotrophic Lateral Sclerosis*.

[54] Sanjak M., Konopacki R., Capasso R. (2004). Dissociation between mechanical and myoelectrical manifestation of muscle fatigue in amyotrophic lateral sclerosis. *Amyotrophic Lateral Sclerosis and Other Motor Neuron Disorders*.

[55] Handy C. R., Krudy C., Boulis N., Federici T. (2011). Pain in amyotrophic lateral sclerosis: a neglected aspect of disease. *Neurology Research International*.

[56] Marchand F., Perretti M., McMahon S. B. (2005). Role of the immune system in chronic pain. *Nature Reviews. Neuroscience*.

[57] Valori C. F., Brambilla L., Martorana F., Rossi D. (2014). The multifaceted role of glial cells in amyotrophic lateral sclerosis. *Cellular and Molecular Life Sciences*.

[58] Tsuda M., Beggs S., Salter M. W., Inoue K. (2013). Microglia and intractable chronic pain. *Glia*.

[59] Brettschneider J., Kurent J., Ludolph A. (2013). Drug therapy for pain in amyotrophic lateral sclerosis or motor neuron disease. *Cochrane Database of Systematic Reviews*.

[60] Attal N., Cruccu G., Baron R. (2010). EFNS guidelines on the pharmacological treatment of neuropathic pain: 2010 revision. *European Journal of Neurology*.

[61] Nijs J., Torres-Cueco R., van Wilgen C. P. (2014). Applying modern pain neuroscience in clinical practice: criteria for the classification of central sensitization pain. *Pain Physician*.

[62] Woolf C. J. (2011). Central sensitization: implications for the diagnosis and treatment of pain. *Pain*.

[63] Woolf C. J. (1983). Evidence for a central component of post-injury pain hypersensitivity. *Nature*.

[64] Ziegler E. A., Magerl W., Meyer R. A., Treede R. D. (1999). Secondary hyperalgesia to punctate mechanical stimuli. *Brain*.

[65] Agosta F., Ferraro P. M., Riva N. (2016). Structural brain correlates of cognitive and behavioral impairment in MND. *Human Brain Mapping*.

[66] Tracey I., Mantyh P. W. (2007). The cerebral signature for pain perception and its modulation. *Neuron*.

[67] Chiò A., Pagani M., Agosta F., Calvo A., Cistaro A., Filippi M. (2014). Neuroimaging in amyotrophic lateral sclerosis: insights into structural and functional changes. *Lancet Neurology*.

[68] Kwak S. (2022). Pain in amyotrophic lateral sclerosis: a narrative review. *Journal of Yeungnam Medical Science*.

[69] Edge R., Mills R., Tennant A., Diggle P. J., Young C. A., TONiC study group (2020). Do pain, anxiety and depression influence quality of life for people with amyotrophic lateral sclerosis/motor neuron disease? A national study reconciling previous conflicting literature. *Journal of Neurology*.

[70] Newrick P. G., Langton-Hewer R. (1985). Pain in motor neuron disease. *Journal of Neurology, Neurosurgery, and Psychiatry*.

[71] D'Ovidio F., d'Errico A., Farina E., Calvo A., Costa G., Chiò A. (2016). Amyotrophic lateral sclerosis incidence and previous prescriptions of drugs for the nervous system. *Neuroepidemiology*.

[72] de Carvalho M., Swash M. (2004). Cramps, muscle pain, and fasciculations: not always benign?. *Neurology*.

[73] Ringel S. P., Murphy J. R., Alderson M. K. (1993). The natural history of amyotrophic lateral sclerosis. *Neurology*.

[74] Wigand B., Schlichte I., Schreiber S. (2022). Characteristics of pain and the burden it causes in patients with amyotrophic lateral sclerosis - a longitudinal study. *Amyotroph Lateral Scler Frontotemporal Degener.*.

[75] Drory V. E., Goltsman E., Reznik J. G., Mosek A., Korczyn A. D. (2001). The value of muscle exercise in patients with amyotrophic lateral sclerosis. *Journal of the Neurological Sciences*.

[76] Pagnini F., Lunetta C., Banfi P. (2012). Pain in amyotrophic lateral sclerosis: a psychological perspective. *Neurological Sciences*.

[77] Adelman E. E., Albert S. M., Rabkin J. G., Del Bene M. L., Tider T., O'Sullivan I. (2004). Disparities in perceptions of distress and burden in ALS patients and family caregivers. *Neurology*.

[78] Simone I. L., Tortelli R., Samarelli V. (2010). Laser evoked potentials in amyotrophic lateral sclerosis. *Journal of the Neurological Sciences*.

[79] Ganzini L., Silveira M. J., Johnston W. S. (2002). Predictors and correlates of interest in assisted suicide in the final month of life among ALS patients in Oregon and Washington. *Journal of Pain and Symptom Management*.

[80] Ganzini L., Johnston W. S., McFarland B. H., Tolle S. W., Lee M. A. (1998). Attitudes of patients with amyotrophic lateral sclerosis and their care givers toward assisted suicide. *The New England Journal of Medicine*.

[81] Ganzini L., Johnston W. S., Silveira M. J. (2002). The final month of life in patients with ALS. *Neurology*.

[82] Abresch R. T., Seyden N. K., Wineinger M. A. (1998). Quality of life. Issues for persons with neuromuscular diseases. *Physical Medicine and Rehabilitation Clinics of North America*.

[83] Mayadev A. S., Weiss M. D., Distad B. J., Krivickas L. S., Carter G. T. (2008). The amyotrophic lateral sclerosis center: a model of multidisciplinary management. *Physical Medicine and Rehabilitation Clinics of North America*.

[84] Carter G. T., Bednar-Butler L. M., Abresch R. T., Ugalde V. O. (1999). Expanding the role of hospice care in amyotrophic lateral sclerosis. *The American Journal of Hospice & Palliative Care*.

[85] Bromberg M. B. (2008). Quality of life in amyotrophic lateral sclerosis. *Physical Medicine and Rehabilitation Clinics of North America*.

[86] Hecht M., Hillemacher T., Gräsel E. (2002). Subjective experience and coping in ALS. *Amyotrophic Lateral Sclerosis and Other Motor Neuron Disorders*.

[87] Meyer T., Funke A., Münch C. (2019). Real world experience of patients with amyotrophic lateral sclerosis (ALS) in the treatment of spasticity using tetrahydrocannabinol: cannabidiol (THC:CBD). *BMC Neurology*.

[88] Åkerblom Y., Jakobsson Larsson B., Zetterberg L., Åsenlöf P. (2020). The multiple faces of pain in motor neuron disease: a qualitative study to inform pain assessment and pain management. *Disability and Rehabilitation*.

[89] Kong Z., Chen P., Jiang J. (2021). Pain characteristics in amyotrophic lateral sclerosis patients and its impact on quality of life: a prospective observational study in a northern city of China. *Annals of Palliative Medicine*.

[90] Melzack R. (1975). The McGill pain questionnaire: major properties and scoring methods. *Pain*.

[91] Warden V., Hurley A. C., Volicer L. (2003). Development and psychometric evaluation of the pain assessment in advanced dementia (PAINAD) scale. *Journal of the American Medical Directors Association*.

[92] Galer B. S., Jensen M. P. (1997). Development and preliminary validation of a pain measure specific to neuropathic pain: the neuropathic pain scale. *Neurology*.

[93] Negri E., Bettaglio R., Demartini L. (2002). Validation of the Italian version of the "neuropathic pain scale" and its clinical applications. *Minerva Anestesiologica*.

[94] Bennett M. (2001). The LANSS pain scale: the Leeds assessment of neuropathic symptoms and signs. *Pain*.

[95] Yucel A., Senocak M., Kocasoy Orhan E., Cimen A., Ertas M. (2004). Results of the Leeds assessment of neuropathic symptoms and signs pain scale in Turkey: a validation study. *The Journal of Pain*.

[96] Krause S. J., Backonja M. M. (2003). Development of a neuropathic pain questionnaire. *The Clinical Journal of Pain*.

[97] Backonja M. M., Krause S. J. (2003). Neuropathic pain questionnaire--short form. *The Clinical Journal of Pain*.

[98] Freynhagen R., Baron R., Gockel U., Tölle T. R. (2006). pain DETECT: a new screening questionnaire to identify neuropathic components in patients with back pain. *Current Medical Research and Opinion*.

[99] Bouhassira D., Attal N., Alchaar H. (2005). Comparison of pain syndromes associated with nervous or somatic lesions and development of a new neuropathic pain diagnostic questionnaire (DN4). *Pain*.

[100] Santos J. G., Brito J. O., de Andrade D. C. (2010). Translation to Portuguese and validation of the Douleur Neuropathique 4 questionnaire. *The Journal of Pain*.

[101] Beswick E., Forbes D., Hassan Z. (2022). A systematic review of non-motor symptom evaluation in clinical trials for amyotrophic lateral sclerosis. *Journal of Neurology*.

[102] Simmons Z., Felgoise S. H., Bremer B. A. (2006). The ALSSQOL. *Neurology*.

[103] Lacomblez L., Bensimon G., Leigh P. N. (1996). A confirmatory dose-ranging study of riluzole in ALS. *Neurology*.

[104] Bensimon G., Lacomblez L., Meininger V. (1994). A controlled trial of riluzole in amyotrophic lateral sclerosis. *The New England Journal of Medicine*.

[105] Lacomblez L., Bensimon G., Leigh P. N., Guillet P., Meininger V. (1996). Dose-ranging study of riluzole in amyotrophic lateral sclerosis. *Lancet*.

[106] WRITING GROUP ON BEHALF OF THE EDARAVONE (MCI-186) ALS 18 STUDY GROUP (2017). Exploratory double-blind, parallel-group, placebo-controlled study of edaravone (MCI-186) in amyotrophic lateral sclerosis (Japan ALS severity classification: grade 3, requiring assistance for eating, excretion or ambulation). *Amyotroph Lateral Scler Frontotemporal Degener*.

[107] Takei K., Takahashi F., Liu S., Tsuda K., Palumbo J. (2017). Post-hoc analysis of randomised, placebo-controlled, double-blind study (MCI186-19) of edaravone (MCI-186) in amyotrophic lateral sclerosis. *Amyotroph Lateral Scler Frontotemporal Degener*.

[108] Oskarsson B., Gendron T. F., Staff N. P. (2018). Amyotrophic lateral sclerosis: an update for 2018. *Mayo Clinic Proceedings*.

[109] van Es M. A., Hardiman O., Chio A. (2017). Amyotrophic lateral sclerosis. *Lancet*.

[110] Rothstein J. D. (2017). Edaravone: a new drug approved for ALS. *Cell*.

[111] Scott A. (2017). Drug therapy: on the treatment trail for ALS. *Nature*.

[112] Chen L., Liu X., Tang L., Zhang N., Fan D. (2016). Long-term use of riluzole could improve the prognosis of sporadic amyotrophic lateral sclerosis patients: a real-world cohort study in China. *Frontiers in Aging Neuroscience*.

[113] Kiernan M. C. (2018). Progress towards therapy in motor neuron disease. *Nature Reviews. Neurology*.

[114] Abe K., Itoyama Y., Sobue G. (2014). Confirmatory double-blind, parallel-group, placebo-controlled study of efficacy and safety of edaravone (MCI-186) in amyotrophic lateral sclerosis patients. *Amyotroph Lateral Scler Frontotemporal Degener*.

[115] Lunetta C., Moglia C., Lizio A. (2020). The Italian multicenter experience with edaravone in amyotrophic lateral sclerosis. *Journal of Neurology*.

[116] Yoshino H., Kimura A. (2006). Investigation of the therapeutic effects of edaravone, a free radical scavenger, on amyotrophic lateral sclerosis (phase II study). *Amyotrophic Lateral Sclerosis*.

[117] Blair H. A. (2023). Tofersen: first approval. *Drugs.*.

[118] Alqallaf A., Cates D. W., Render K. P., Patel K. A. (2024). Sodium phenylbutyrate and taurursodiol: a new therapeutic option for the treatment of amyotrophic lateral sclerosis. *The Annals of Pharmacotherapy*.

[119] Meyer T., Schumann P., Weydt P. (2023). Neurofilament light-chain response during therapy with antisense oligonucleotide tofersen inSOD1‐relatedALS: treatment experience in clinical practice. *Muscle & Nerve*.

[120] Steinacker P., Feneberg E., Weishaupt J. (2015). Neurofilaments in the diagnosis of motoneuron diseases: a prospective study on 455 patients. *Journal of Neurology, Neurosurgery, and Psychiatry*.

[121] Paganoni S., Hendrix S., Dickson S. P. (2021). Long-term survival of participants in the CENTAUR trial of sodium phenylbutyrate-taurursodiol in amyotrophic lateral sclerosis. *Muscle Nerve*.

[122] Hobson E. V., McDermott C. J. (2016). Supportive and symptomatic management of amyotrophic lateral sclerosis. *Nature Reviews. Neurology*.

[123] Hardiman O., Al-Chalabi A., Chio A. (2017). Amyotrophic lateral sclerosis. *Nature Reviews Disease Primers*.

[124] Soriani M. H., Desnuelle C. (2017). Care management in amyotrophic lateral sclerosis. *Revue Neurologique (Paris)*.

[125] Chiò A., Silani V., Italian ALS Study Group (2001). Amyotrophic lateral sclerosis care in Italy: a nationwide study in neurological centers. *Journal of the Neurological Sciences*.

[126] O'Brien T., Kelly M., Saunders C. (1992). Motor neurone disease: a hospice perspective. *BMJ*.

[127] Oliver D. (1998). Opioid medication in the palliative care of motor neurone disease. *Palliative Medicine*.

[128] Oliver D. (1996). The quality of care and symptom control--the effects on the terminal phase of ALS/MND. *Journal of the Neurological Sciences.*.

[129] Neudert C., Oliver D., Wasner M., Borasio G. D. (2001). The course of the terminal phase in patients with amyotrophic lateral sclerosis. *Journal of Neurology*.

[130] Oskarsson B., Moore D., Mozaffar T. (2018). Mexiletine for muscle cramps in amyotrophic lateral sclerosis: a randomized, double-blind crossover trial. *Muscle and Nerve*.

[131] Adiao K. J. B., Espiritu A. I., Bagnas M. A. C. (2020). Efficacy and safety of mexiletine in amyotrophic lateral sclerosis: a systematic review of randomized controlled trials. *Neurodegenerative Disease Management*.

[132] Hogan D. B. (2015). Quinine and leg cramps. *Canadian Medical Association Journal*.

[133] Baldinger R., Katzberg H. D., Weber M., Cochrane Neuromuscular Group (2012). Treatment for cramps in amyotrophic lateral sclerosis/motor neuron disease. *Cochrane Database of Systematic Reviews*.

[134] Houstoun M., Reichman M. E., Graham D. J. (2014). Use of an active surveillance system by the FDA to observe patterns of quinine sulfate use and adverse hematologic outcomes in CMS Medicare data. *Pharmacoepidemiology and Drug Safety*.

[135] El-Tawil S., Al Musa T., Valli H. (2015). Quinine for muscle cramps. *Cochrane Database of Systematic Reviews*.

[136] Coffey J. R., Cahill D., Steers W. (1993). Intrathecal baclofen for intractable spasticity of spinal origin: results of a long-term multicenter study. *Journal of Neurosurgery*.

[137] Kita M., Goodkin D. E. (2000). Drugs used to treat spasticity. *Drugs*.

[138] Marquardt G., Seifert V. (2002). Use of intrathecal baclofen for treatment of spasticity in amyotrophic lateral sclerosis. *Journal of Neurology, Neurosurgery, and Psychiatry*.

[139] McClelland S., Bethoux F. A., Boulis N. M. (2008). Intrathecal baclofen for spasticity-related pain in amyotrophic lateral sclerosis: efficacy and factors associated with pain relief. *Muscle and Nerve*.

[140] Ashworth N. L., Satkunam L. E., Deforge D., Cochrane Neuromuscular Group (2012). Treatment for spasticity in amyotrophic lateral sclerosis/motor neuron disease. *Cochrane Database of Systematic Reviews*.

[141] Green S., Buchbinder R., Hetrick S., Cochrane Musculoskeletal Group (2005). Acupuncture for shoulder pain. *Cochrane Database of Systematic Reviews*.

[142] Maggiani A., Tremolizzo L., Della Valentina A. (2016). Osteopathic manual treatment for amyotrophic lateral sclerosis: a feasibility pilot study. *The Open Neurology Journal*.

[143] Mikuriya T. H. (1969). Marijuana in medicine: past, present and future. *California Medicine*.

[144] Andre C. M., Hausman J. F., Guerriero G. (2016). Cannabis sativa: the plant of the thousand and one molecules. *Frontiers in Plant Science*.

[145] Touw M. (1981). The religious and medicinal uses of Cannabis in China, India and Tibet. *Journal of Psychoactive Drugs*.

[146] Friedman D., Devinsky O. (2016). Cannabinoids in the treatment of epilepsy. *The New England Journal of Medicine*.

[147] Cerdá M., Wall M., Keyes K. M., Galea S., Hasin D. (2012). Medical marijuana laws in 50 states: investigating the relationship between state legalization of medical marijuana and marijuana use, abuse and dependence. *Drug and Alcohol Dependence*.

[148] Sun Y., Bennett A. (2007). Cannabinoids: a new group of agonists of PPARs. *PPAR Research*.

[149] Alves P., Amaral C., Teixeira N., Correia-da-Silva G. (2020). Cannabis sativa: much more beyond *Δ*^9^-tetrahydrocannabinol. *Pharmacological Research*.

[150] Maroon J., Bost J. (2018). Review of the neurological benefits of phytocannabinoids. *Surgical Neurology International*.

[151] Gaoni Y., Mechoulam R. (1964). Isolation, structure, and partial synthesis of an active constituent of hashish. *Journal of the American Chemical Society*.

[152] Gaoni Y., Mechoulam R. (1971). The isolation and structure of delta-1-tetrahydrocannabinol and other neutral cannabinoids from hashish. *Journal of the American Chemical Society*.

[153] Turner S. E., Williams C. M., Iversen L., Whalley B. J. (2017). Molecular pharmacology of phytocannabinoids. *Progress in the Chemistry of Organic Natural Products*.

[154] Bhattacharyya S., Morrison P. D., Fusar-Poli P. (2010). Opposite effects of delta-9-tetrahydrocannabinol and cannabidiol on human brain function and psychopathology. *Neuropsychopharmacology*.

[155] Zuardi A. W., Shirakawa I., Finkelfarb E., Karniol I. G. (1982). Action of cannabidiol on the anxiety and other effects produced by delta 9-THC in normal subjects. *Psychopharmacology*.

[156] Smith D. E. (1998). Review of the American Medical Association council on scientific affairs report on medical marijuana. *Journal of Psychoactive Drugs*.

[157] Martin B. R., Compton D. R., Thomas B. F. (1991). Behavioral, biochemical, and molecular modeling evaluations of cannabinoid analogs. *Pharmacology, Biochemistry, and Behavior*.

[158] Pagano C., Navarra G., Coppola L., Avilia G., Bifulco M., Laezza C. (2022). Cannabinoids: therapeutic use in clinical practice. *International Journal of Molecular Sciences*.

[159] Mücke M., Phillips T., Radbruch L., Petzke F., Häuser W. (2018). Cannabis-based medicines for chronic neuropathic pain in adults. *Cochrane Database of Systematic Reviews*.

[160] Fernández-Ruiz J., Galve-Roperh I., Sagredo O., Guzmán M. (2020). Possible therapeutic applications of cannabis in the neuropsychopharmacology field. *European Neuropsychopharmacology*.

[161] Devane W. A., Breuer A., Sheskin T., Järbe T. U., Eisen M. S., Mechoulam R. (1992). A novel probe for the cannabinoid receptor. *Journal of Medicinal Chemistry*.

[162] Lambert D. M., Fowler C. J. (2005). The endocannabinoid system: drug targets, lead compounds, and potential therapeutic applications. *Journal of Medicinal Chemistry*.

[163] Katona I., Freund T. F. (2012). Multiple functions of endocannabinoid signaling in the brain. *Annual Review of Neuroscience*.

[164] Mechoulam R., Ben-Shabat S., Hanus L. (1995). Identification of an endogenous 2-monoglyceride, present in canine gut, that binds to cannabinoid receptors. *Biochemical Pharmacology*.

[165] Piomelli D. (2003). The molecular logic of endocannabinoid signalling. *Nature Reviews. Neuroscience*.

[166] Curran H. V., Freeman T. P., Mokrysz C., Lewis D. A., Morgan C. J. A., Parsons L. H. (2016). Keep off the grass? Cannabis, cognition and addiction. *Nature Reviews. Neuroscience*.

[167] Onaivi E. S. (2007). Neuropsychobiological evidence for the functional presence and expression of cannabinoid CB2 receptors in the brain. *Neuropsychobiology*.

[168] Maresz K., Carrier E. J., Ponomarev E. D., Hillard C. J., Dittel B. N. (2005). Modulation of the cannabinoid CB2 receptor in microglial cells in response to inflammatory stimuli. *Journal of Neurochemistry*.

[169] Carlisle S. J., Marciano-Cabral F., Staab A., Ludwick C., Cabral G. A. (2002). Differential expression of the CB2 cannabinoid receptor by rodent macrophages and macrophage-like cells in relation to cell activation. *International Immunopharmacology*.

[170] Dheen S. T., Kaur C., Ling E. A. (2007). Microglial activation and its implications in the brain diseases. *Current Medicinal Chemistry*.

[171] Sheng W. S., Hu S., Min X., Cabral G. A., Lokensgard J. R., Peterson P. K. (2005). Synthetic cannabinoid WIN55,212-2 inhibits generation of inflammatory mediators by IL-1beta-stimulated human astrocytes. *Glia*.

[172] Stella N. (2010). Cannabinoid and cannabinoid-like receptors in microglia, astrocytes, and astrocytomas. *Glia*.

[173] Cencioni M. T., Chiurchiù V., Catanzaro G. (2010). Anandamide suppresses proliferation and cytokine release from primary human T-lymphocytes mainly via CB2 receptors. *PLoS One*.

[174] Pasquariello N., Catanzaro G., Marzano V. (2009). Characterization of the endocannabinoid system in human neuronal cells and proteomic analysis of anandamide-induced apoptosis. *Journal of Biological Chemistry*.

[175] Pandey R., Mousawy K., Nagarkatti M., Nagarkatti P. (2009). Endocannabinoids and immune regulation. *Pharmacological Research*.

[176] Parolaro D., Massi P., Rubino T., Monti E. (2002). Endocannabinoids in the immune system and cancer. *Prostaglandins, Leukotrienes, and Essential Fatty Acids*.

[177] Espejo-Porras F., García-Toscano L., Rodríguez-Cueto C., Santos-García I., de Lago E., Fernandez-Ruiz J. (2019). Targeting glial cannabinoid CB_2_ receptors to delay the progression of the pathological phenotype in TDP-43 (A315T) transgenic mice, a model of amyotrophic lateral sclerosis. *British Journal of Pharmacology*.

[178] Urbi B., Owusu M. A., Hughes I., Katz M., Broadley S., Sabet A. (2019). Effects of cannabinoids in amyotrophic lateral sclerosis (ALS) murine models: a systematic review and meta-analysis. *Journal of Neurochemistry*.

[179] Hampson A. J., Grimaldi M., Axelrod J., Wink D. (1998). Cannabidiol and (-)delta9-tetrahydrocannabinol are neuroprotective antioxidants. *Proceedings of the National Academy of Sciences of the United States of America*.

[180] Nagayama T., Sinor A. D., Simon R. P. (1999). Cannabinoids and neuroprotection in global and focal cerebral ischemia and in neuronal cultures. *The Journal of Neuroscience*.

[181] Eshhar N., Striem S., Biegon A. (1993). HU-211, a non-psychotropic cannabinoid, rescues cortical neurones from excitatory amino acid toxicity in culture. *Neuroreport*.

[182] Basavarajappa B. S., Shivakumar M., Joshi V., Subbanna S. (2017). Endocannabinoid system in neurodegenerative disorders. *Journal of Neurochemistry*.

[183] Di Marzo V., Stella N., Zimmer A. (2015). Endocannabinoid signalling and the deteriorating brain. *Nature Reviews Neuroscience*.

[184] Ren S. Y., Wang Z. Z., Zhang Y., Chen N. H. (2020). Potential application of endocannabinoid system agents in neuropsychiatric and neurodegenerative diseases-focusing on FAAH/MAGL inhibitors. *Acta Pharmacologica Sinica*.

[185] Croxford J. L. (2003). Therapeutic potential of cannabinoids in CNS disease. *CNS Drugs*.

[186] Adermark L., Lovinger D. M. (2007). Retrograde endocannabinoid signaling at striatal synapses requires a regulated postsynaptic release step. *Proceedings of the National Academy of Sciences of the United States of America*.

[187] Adermark L., Talani G., Lovinger D. M. (2009). Endocannabinoid-dependent plasticity at GABAergic and glutamatergic synapses in the striatum is regulated by synaptic activity. *European Journal of Neuroscience*.

[188] Riegel A. C., Lupica C. R. (2004). Independent presynaptic and postsynaptic mechanisms regulate endocannabinoid signaling at multiple synapses in the ventral tegmental area. *The Journal of Neuroscience*.

[189] Scotter E. L., Abood M. E., Glass M. (2010). The endocannabinoid system as a target for the treatment of neurodegenerative disease. *British Journal of Pharmacology*.

[190] Burgess R. W., Cox G. A., Seburn K. L. (2010). Neuromuscular disease models and analysis. *Methods in Molecular Biology*.

[191] Weiss M. D., Carter G. T. (2008). Preface: the state of the science of all motor neuron diseases. *Physical Medicine and Rehabilitation Clinics of North America*.

[192] Wallace M. J., Martin B. R., DeLorenzo R. J. (2002). Evidence for a physiological role of endocannabinoids in the modulation of seizure threshold and severity. *European Journal of Pharmacology*.

[193] Baker D., Pryce G., Giovannoni G., Thompson A. J. (2003). The therapeutic potential of cannabis. *Lancet Neurology*.

[194] Richardson J. D., Kilo S., Hargreaves K. M. (1998). Cannabinoids reduce hyperalgesia and inflammation via interaction with peripheral CB1 receptors. *Pain*.

[195] Mechoulam R., Panikashvili D., Shohami E. (2002). Cannabinoids and brain injury: therapeutic implications. *Trends in Molecular Medicine*.

[196] Zhao P., Leonoudakis D., Abood M. E., Beattie E. C. (2010). Cannabinoid receptor activation reduces TNFalpha-induced surface localization of AMPAR-type glutamate receptors and excitotoxicity. *Neuropharmacology*.

[197] Elikkottil J., Gupta P., Gupta K. (2010). The analgesic potential of cannabinoids (Journal of Opioid Management (2009), 5, 6,(341-357)). *Journal of Opioid Management*.

[198] Mato S., Alberdi E., Ledent C., Watanabe M., Matute C. (2009). CB1 cannabinoid receptor-dependent and -independent inhibition of depolarization-induced calcium influx in oligodendrocytes. *Glia*.

[199] Anand P., Whiteside G., Fowler C. J., Hohmann A. G. (2009). Targeting CB2 receptors and the endocannabinoid system for the treatment of pain. *Brain Research Reviews*.

[200] Meng I. D., Manning B. H., Martin W. J., Fields H. L. (1998). An analgesia circuit activated by cannabinoids. *Nature*.

[201] McCarberg B. H., Barkin R. L. (2007). The future of cannabinoids as analgesic agents: a pharmacologic, pharmacokinetic, and pharmacodynamic overview. *American Journal of Therapeutics*.

[202] Reche I., Fuentes J. A., Ruiz-Gayo M. (1996). Potentiation of delta 9-tetrahydrocannabinol-induced analgesia by morphine in mice: involvement of mu- and kappa-opioid receptors. *European Journal of Pharmacology*.

[203] Yesilyurt O., Dogrul A., Gul H. (2003). Topical cannabinoid enhances topical morphine antinociception. *Pain*.

[204] Finn D. P., Beckett S. R., Roe C. H. (2004). Effects of coadministration of cannabinoids and morphine on nociceptive behaviour, brain monoamines and HPA axis activity in a rat model of persistent pain. *The European Journal of Neuroscience*.

[205] Tham S. M., Angus J. A., Tudor E. M., Wright C. E. (2005). Synergistic and additive interactions of the cannabinoid agonist CP55,940 with mu opioid receptor and alpha 2-adrenoceptor agonists in acute pain models in mice. *British Journal of Pharmacology*.

[206] Cooper Z. D., Bedi G., Ramesh D., Balter R., Comer S. D., Haney M. (2018). Impact of co-administration of oxycodone and smoked cannabis on analgesia and abuse liability. *Neuropsychopharmacology*.

[207] Ibrahim M. M., Porreca F., Lai J. (2005). CB2 cannabinoid receptor activation produces antinociception by stimulating peripheral release of endogenous opioids. *Proceedings of the National Academy of Sciences of the United States of America*.

[208] Wilsey B., Marcotte T. D., Deutsch R., Zhao H., Prasad H., Phan A. (2016). An exploratory human laboratory experiment evaluating vaporized cannabis in the treatment of neuropathic pain from spinal cord injury and disease. *The Journal of Pain*.

[209] Poli P., Crestani F., Salvadori C., Valenti I., Sannino C. (2018). Medical cannabis in patients with chronic pain: effect on pain relief, pain disability, and psychological aspects. A prospective non randomized single arm clinical trial. *Clinical Therapeutics*.

[210] Liang D., Bao Y., Wallace M., Grant I., Shi Y. (2018). Medical cannabis legalization and opioid prescriptions: evidence on US Medicaid enrollees during 1993-2014. *Addiction*.

[211] Bellnier T., Brown G. W., Ortega T. R. (2018). Preliminary evaluation of the efficacy, safety, and costs associated with the treatment of chronic pain with medical cannabis. *Mental Health & Wellness Clinic*.

[212] Cox M. L., Haller V. L., Welch S. P. (2007). Synergy between *Δ*9-tetrahydrocannabinol and morphine in the arthritic rat. *European Journal of Pharmacology*.

[213] Cleveland D. W., Rothstein J. D. (2001). From Charcot to Lou Gehrig: deciphering selective motor neuron death in ALS. *Nature Reviews. Neuroscience*.

[214] Witting A., Weydt P., Hong S., Kliot M., Moller T., Stella N. (2004). Endocannabinoids accumulate in spinal cord of SOD1 G93A transgenic mice. *Journal of Neurochemistry*.

[215] Shoemaker J. L., Seely K. A., Reed R. L., Crow J. P., Prather P. L. (2007). The CB2 cannabinoid agonist AM-1241 prolongs survival in a transgenic mouse model of amyotrophic lateral sclerosis when initiated at symptom onset. *Journal of Neurochemistry*.

[216] Espejo-Porras F., Piscitelli F., Verde R. (2015). Changes in the endocannabinoid signaling system in CNS structures of TDP-43 transgenic mice: relevance for a neuroprotective therapy in TDP-43-related disorders. *Journal of Neuroimmune Pharmacology*.

[217] Yang C., Wang H., Qiao T. (2014). Partial loss of TDP-43 function causes phenotypes of amyotrophic lateral sclerosis. *Proceedings of the National Academy of Sciences of the United States of America*.

[218] Barmada S. J., Skibinski G., Korb E., Rao E. J., Wu J. Y., Finkbeiner S. (2010). Cytoplasmic mislocalization of TDP-43 is toxic to neurons and enhanced by a mutation associated with familial amyotrophic lateral sclerosis. *The Journal of Neuroscience*.

[219] Bilsland L. G., Dick J. R., Pryce G. (2006). Increasing cannabinoid levels by pharmacological and genetic manipulation delay disease progression in SOD1 mice. *The FASEB Journal*.

[220] Kim K., Moore D. H., Makriyannis A., Abood M. E. (2006). AM1241, a cannabinoid CB2 receptor selective compound, delays disease progression in a mouse model of amyotrophic lateral sclerosis. *European Journal of Pharmacology*.

[221] Raman C., McAllister S. D., Rizvi G., Patel S. G., Moore D. H., Abood M. E. (2004). Amyotrophic lateral sclerosis: delayed disease progression in mice by treatment with a cannabinoid. *Amyotrophic Lateral Sclerosis and Other Motor Neuron Disorders*.

[222] Abood M. E., Rizvi G., Sallapudi N., McAllister S. D. (2001). Activation of the CB1 cannabinoid receptor protects cultured mouse spinal neurons against excitotoxicity. *Neuroscience Letters*.

[223] Yiangou Y., Facer P., Durrenberger P. (2006). COX-2, CB2 and P2X7-immunoreactivities are increased in activated microglial cells/macrophages of multiple sclerosis and amyotrophic lateral sclerosis spinal cord. *BMC Neurology*.

[224] Chiurchiù V., Leuti A., Maccarrone M. (2015). Cannabinoid signaling and neuroinflammatory diseases: a melting pot for the regulation of brain immune responses. *Journal of Neuroimmune Pharmacology*.

[225] Gerdeman G. L., Lovinger D. M. (2003). Emerging roles for endocannabinoids in long-term synaptic plasticity. *British Journal of Pharmacology*.

[226] Robson P. (2005). Human studies of cannabinoids and medicinal cannabis. *Handbook of Experimental Pharmacology*.

[227] Carter G. T., Abood M. E., Aggarwal S. K., Weiss M. D. (2010). Cannabis and amyotrophic lateral sclerosis: hypothetical and practical applications, and a call for clinical trials. *The American Journal of Hospice & Palliative Care*.

[228] Amtmann D., Weydt P., Johnson K. L., Jensen M. P., Carter G. T. (2004). Survey of cannabis use in patients with amyotrophic lateral sclerosis. *The American Journal of Hospice & Palliative Care*.

[229] Carter G. T., Rosen B. S. (2001). Marijuana in the management of amyotrophic lateral sclerosis. *The American Journal of Hospice & Palliative Care*.

[230] Lakhan S. E., Rowland M. (2009). Whole plant cannabis extracts in the treatment of spasticity in multiple sclerosis: a systematic review. *BMC Neurology*.

[231] Carter G. T., Johnson E. R., Bonekat H. W., Lieberman J. S. (1992). Laryngeal diversion in the treatment of intractable aspiration in motor neuron disease. *Archives of Physical Medicine and Rehabilitation*.

[232] Collamati A., Martone A. M., Poscia A. (2016). Anticholinergic drugs and negative outcomes in the older population: from biological plausibility to clinical evidence. *Aging Clinical and Experimental Research*.

[233] Rosenfeld J., Ellis A. (2008). Nutrition and dietary supplements in motor neuron disease. *Physical Medicine and Rehabilitation Clinics of North America*.

[234] Hammond S., Erridge S., Mangal N., Pacchetti B., Sodergren M. H. (2021). The effect of cannabis-based medicine in the treatment of cachexia: a systematic review and meta-analysis. *Cannabis and Cannabinoid Research*.

[235] Mazier W., Saucisse N., Gatta-Cherifi B., Cota D. (2015). The endocannabinoid system: pivotal orchestrator of obesity and metabolic disease. *Trends in Endocrinology and Metabolism*.

[236] Grotenhermen F. (2003). Pharmacokinetics and pharmacodynamics of cannabinoids. *Clinical Pharmacokinetics*.

[237] Williams S. J., Hartley J. P., Graham J. D. (1976). Bronchodilator effect of delta1-tetrahydrocannabinol administered by aerosol of asthmatic patients. *Thorax*.

[238] Hartley J. P., Nogrady S. G., Seaton A. (1978). Bronchodilator effect of delta1-tetrahydrocannabinol. *British Journal of Clinical Pharmacology*.

[239] Tashkin D. P., Shapiro B. J., Frank I. M. (1973). Acute pulmonary physiologic effects of smoked marijuana and oral *Δ*9-tetrahydrocannabinol in healthy young men. *The New England Journal of Medicine*.

[240] McGeer E. G., McGeer P. L. (2005). Pharmacologic approaches to the treatment of amyotrophic lateral sclerosis. *BioDrugs*.

[241] Cui F., Zhu W., Zhou Z. (2015). Frequency and risk factor analysis of cognitive and anxiety-depressive disorders in patients with amyotrophic lateral sclerosis/motor neuron disease. *Neuropsychiatric Disease and Treatment*.

[242] Rabkin J. G., Albert S. M., Del Bene M. L. (2005). Prevalence of depressive disorders and change over time in late-stage ALS. *Neurology*.

[243] Kurt A., Nijboer F., Matuz T., Kübler A. (2007). Depression and anxiety in individuals with amyotrophic lateral sclerosis. *CNS Drugs*.

[244] Lutz B., Marsicano G., Maldonado R., Hillard C. J. (2015). The endocannabinoid system in guarding against fear, anxiety and stress. *Nature Reviews Neuroscience*.

[245] Nunberg H., Kilmer B., Pacula R. L., Burgdorf J. (2011). An analysis of applicants presenting to a medical marijuana specialty practice in California. *Journal of Drug Policy Analysis*.

[246] Brindisi M., Maramai S., Gemma S. (2016). Development and pharmacological characterization of selective blockers of 2-arachidonoyl glycerol degradation with efficacy in rodent models of multiple sclerosis and pain. *Journal of Medicinal Chemistry*.

[247] Butini S., Brindisi M., Gemma S. (2012). Discovery of potent inhibitors of human and mouse fatty acid amide hydrolases. *Journal of Medicinal Chemistry*.

[248] Grillo A., Fezza F., Chemi G. (2021). Selective fatty acid amide hydrolase inhibitors as potential novel antiepileptic agents. *ACS Chemical Neuroscience*.

[249] Brindisi M., Borrelli G., Brogi S. (2018). Development of potent inhibitors of fatty acid amide hydrolase useful for the treatment of neuropathic pain. *ChemMedChem*.

[250] van Egmond N., Straub V. M., van der Stelt M. (2021). Targeting endocannabinoid signaling: FAAH and MAG lipase inhibitors. *Annual Review of Pharmacology and Toxicology*.

[251] Anderson W. B., Gould M. J., Torres R. D., Mitchell V. A., Vaughan C. W. (2014). Actions of the dual FAAH/MAGL inhibitor JZL195 in a murine inflammatory pain model. *Neuropharmacology*.

[252] Ramesh D., Gamage T. F., Vanuytsel T. (2013). Dual inhibition of endocannabinoid catabolic enzymes produces enhanced antiwithdrawal effects in morphine-dependent mice. *Neuropsychopharmacology*.

[253] Martín-Sánchez E., Furukawa T. A., Taylor J., Martin J. L. (2009). Systematic review and meta-analysis of cannabis treatment for chronic pain. *Pain Medicine*.

[254] Wilkerson J. L., Ghosh S., Mustafa M. (2017). The endocannabinoid hydrolysis inhibitor SA-57: intrinsic antinociceptive effects, augmented morphine-induced antinociception, and attenuated heroin seeking behavior in mice. *Neuropharmacology*.

[255] Talman P., Duong T., Vucic S. (2016). Identification and outcomes of clinical phenotypes in amyotrophic lateral sclerosis/motor neuron disease: Australian National Motor Neuron Disease observational cohort. *BMJ Open*.

[256] Russo E., Guy G. W. (2006). A tale of two cannabinoids: the therapeutic rationale for combining tetrahydrocannabinol and cannabidiol. *Medical Hypotheses*.

[257] Zettl U. K., Rommer P., Hipp P., Patejdl R. (2016). Evidence for the efficacy and effectiveness of THC-CBD oromucosal spray in symptom management of patients with spasticity due to multiple sclerosis. *Therapeutic Advances in Neurological Disorders*.

[258] Legare C. A., Raup-Konsavage W. M., Vrana K. E. (2022). Therapeutic potential of cannabis, cannabidiol, and cannabinoid-based pharmaceuticals. *Pharmacology*.

[259] Collin C., Davies P., Mutiboko I. K., Ratcliffe S., for the Sativex Spasticity in MS Study Group (2007). Randomized controlled trial of cannabis-based medicine in spasticity caused by multiple sclerosis. *European Journal of Neurology*.

[260] Meinck H. M., Schönle P. W., Conrad B. (1989). Effect of cannabinoids on spasticity and ataxia in multiple sclerosis. *Journal of Neurology*.

[261] Greenberg H. S., Werness S. A., Pugh J. E., Andrus R. O., Anderson D. J., Domino E. F. (1994). Short-term effects of smoking marijuana on balance in patients with multiple sclerosis and normal volunteers. *Clinical Pharmacology and Therapeutics*.

[262] Fitzgerald P. B., Williams S., Daskalakis Z. J. (2009). A transcranial magnetic stimulation study of the effects of cannabis use on motor cortical inhibition and excitability. *Neuropsychopharmacology*.

[263] Wade D. T., Makela P., Robson P., House H., Bateman C. (2004). Do cannabis-based medicinal extracts have general or specific effects on symptoms in multiple sclerosis? A double-blind, randomized, placebo-controlled study on 160 patients. *Multiple Sclerosis*.

[264] Patti F., Messina S., Solaro C. (2016). Efficacy and safety of cannabinoid oromucosal spray for multiple sclerosis spasticity. *Journal of Neurology, Neurosurgery, and Psychiatry*.

[265] Riva N., Mora G., Sorarù G. (2019). Safety and efficacy of nabiximols on spasticity symptoms in patients with motor neuron disease (CANALS): a multicentre, double-blind, randomised, placebo-controlled, phase 2 trial. *Lancet Neurology*.

[266] Jensen M. P., McFarland C. A. (1993). Increasing the reliability and validity of pain intensity measurement in chronic pain patients. *Pain*.

[267] Goulet J. L., Kerns R. D., Bair M. (2016). The musculoskeletal diagnosis cohort: examining pain and pain care among veterans. *Pain*.

[268] Atkinson M. J., Kumar R., Cappelleri J. C., Hass S. L. (2005). Hierarchical construct validity of the treatment satisfaction questionnaire for medication (TSQM version II) among outpatient pharmacy consumers. *Value in Health*.

[269] Newman Z., Malik P., Wu T. Y., Ochoa C., Watsa N., Lindgren C. (2007). Endocannabinoids mediate muscarine-induced synaptic depression at the vertebrate neuromuscular junction. *European Journal of Neuroscience*.

[270] Zhao P., Ignacio S., Beattie E. C., Abood M. E. (2008). Altered presymptomatic AMPA and cannabinoid receptor trafficking in motor neurons of ALS model mice: implications for excitotoxicity. *European Journal of Neuroscience*.

[271] Weber M., Goldman B., Truniger S. (2010). Tetrahydrocannabinol (THC) for cramps in amyotrophic lateral sclerosis: a randomised, double-blind crossover trial. *Journal of Neurology, Neurosurgery, and Psychiatry*.

[272] Talbott E. O., Malek A. M., Lacomis D. (2016). The epidemiology of amyotrophic lateral sclerosis. *Handbook of Clinical Neurology*.

[273] Mehta P., Kaye W., Raymond J. (2018). Prevalence of amyotrophic lateral sclerosis-United States, 2015. *MMWR. Morbidity and Mortality Weekly Report*.

[274] Lacroix C., Guilhaumou R., Micallef J., Bruneteau G., Desnuelle C., Blin O. (2023). Cannabis for the treatment of amyotrophic lateral sclerosis: what is the patients' view?. *Revue Neurologique (Paris)*.

[275] Mechoulam R., Devane W. A., Breuer A., Zahalka J. (1991). A random walk through a cannabis field. *Pharmacology, Biochemistry, and Behavior*.

[276] Russell C., Rueda S., Room R., Tyndall M., Fischer B. (2018). Routes of administration for cannabis use - basic prevalence and related health outcomes: a scoping review and synthesis. *The International Journal on Drug Policy*.

[277] Gurley R. J., Aranow R., Katz M. (1998). Medicinal marijuana: a comprehensive review. *Journal of Psychoactive Drugs*.

[278] Loflin M., Earleywine M. (2014). A new method of cannabis ingestion: the dangers of dabs?. *Addictive Behaviors*.

[279] Steigerwald S., Wong P. O., Khorasani A., Keyhani S. (2018). The form and content of cannabis products in the United States. *Journal of General Internal Medicine*.

[280] Borodovsky J. T., Lee D. C., Crosier B. S., Gabrielli J. L., Sargent J. D., Budney A. J. (2017). U.S. cannabis legalization and use of vaping and edible products among youth. *Drug Alcohol Depend*.

[281] Cuttler C., Mischley L. K., Sexton M. (2016). Sex differences in cannabis use and effects: a cross-sectional survey of cannabis users. *Cannabis and Cannabinoid Research*.

[282] Haug N. A., Padula C. B., Sottile J. E., Vandrey R., Heinz A. J., Bonn-Miller M. O. (2017). Cannabis use patterns and motives: a comparison of younger, middle-aged, and older medical cannabis dispensary patients. *Addictive Behaviors*.

[283] Lankenau S. E., Fedorova E. V., Reed M., Schrager S. M., Iverson E., Wong C. F. (2017). Marijuana practices and patterns of use among young adult medical marijuana patients and non-patient marijuana users. *Drug and Alcohol Dependence*.

[284] Newmeyer M. N., Swortwood M. J., Abulseoud O. A., Huestis M. A. (2017). Subjective and physiological effects, and expired carbon monoxide concentrations in frequent and occasional cannabis smokers following smoked, vaporized, and oral cannabis administration. *Drug and Alcohol Dependence*.

[285] Jones R. T., Benowitz N. L., Herning R. I. (1981). Clinical relevance of cannabis tolerance and dependence. *Journal of Clinical Pharmacology*.

[286] Voth E. A., Schwartz R. H. (1997). Medicinal applications of delta-9-tetrahydrocannabinol and marijuana. *Annals of Internal Medicine*.

[287] Cranford J. A., Bohnert K. M., Perron B. E., Bourque C., Ilgen M. (2016). Prevalence and correlates of "vaping" as a route of cannabis administration in medical cannabis patients. *Drug and Alcohol Dependence*.

[288] Morean M. E., Lipshie N., Josephson M., Foster D. (2017). Predictors of adult E-cigarette users vaporizing cannabis using E-cigarettes and vape-pens. *Substance Use & Misuse*.

[289] Lee D. C., Crosier B. S., Borodovsky J. T., Sargent J. D., Budney A. J. (2016). Online survey characterizing vaporizer use among cannabis users. *Drug and Alcohol Dependence*.

[290] Loflin M., Earleywine M. (2015). No smoke, no fire: what the initial literature suggests regarding vapourized cannabis and respiratory risk. *Canadian Journal of Respiratory Therapy*.

[291] Todd T. (2018). The benefits of marijuana legalization and regulation. *Berkeley Journal of Criminal Law*.

[292] Agurell S., Halldin M., Lindgren J. E. (1986). Pharmacokinetics and metabolism of delta 1-tetrahydrocannabinol and other cannabinoids with emphasis on man. *Pharmacological Reviews*.

[293] Matthias P., Tashkin D. P., Marques-Magallanes J. A., Wilkins J. N., Simmons M. S. (1997). Effects of varying marijuana potency on deposition of tar and delta9-THC in the lung during smoking. *Pharmacology, Biochemistry, and Behavior*.

[294] Carter G. T., Weydt P., Kyashna-Tocha M., Abrams D. I. (2004). Medicinal cannabis: rational guidelines for dosing. *IDrugs*.

[295] Cohen K., Weizman A., Weinstein A. (2019). Positive and negative effects of cannabis and cannabinoids on health. *Clinical Pharmacology and Therapeutics*.

[296] Gibbs M., Winsper C., Marwaha S., Gilbert E., Broome M., Singh S. P. (2015). Cannabis use and mania symptoms: a systematic review and meta-analysis. *Journal of Affective Disorders*.

[297] Horwood L. J., Fergusson D. M., Coffey C. (2012). Cannabis and depression: an integrative data analysis of four Australasian cohorts. *Drug and Alcohol Dependence*.

[298] van Os J., Bak M., Hanssen M., Bijl R. V., de Graaf R., Verdoux H. (2002). Cannabis use and psychosis: a longitudinal population-based study. *American Journal of Epidemiology*.

[299] Lorenzetti V., Solowij N., Yücel M. (2016). The role of cannabinoids in neuroanatomic alterations in cannabis users. *Biological Psychiatry*.

[300] Tashkin D. P., Simmons M. S., Tseng C. H. (2012). Impact of changes in regular use of marijuana and/or tobacco on chronic bronchitis. *Chronic Obstructive Pulmonary Disease*.

[301] Clark P. A., Capuzzi K., Fick C. (2011). Medical marijuana: medical necessity versus political agenda. *Medical Science Monitor*.

[302] Verma R., Hoda F., Arshad M. (2021). Cannabis, a miracle drug with polyvalent therapeutic utility: preclinical and clinical-based evidence. *Medical Cannabis and Cannabinoids*.

