# Supplementary material for: Amyotrophic Lateral Sclerosis and Pain: A Narrative Review from Pain Assessment to Therapy
Source: Behav Neurol. 2024 Mar 16;2024:1228194. doi: 10.1155/2024/1228194 (PMC10960655; doi:10.1155/2024/1228194)
Supplement: Supplementary Materials — Table 1: pain assessment tool. [file 1228194.f1.docx]

| PAIN ASSESSMENT TOOL | DESCRIPTION | TYPE OF EVALUATION | SCORE RANGE | PAIN RATING | VALIDITY/ RELIABILITY | INTENDED USE | VALIDATED IN THE ALS POPULATION |
| --- | --- | --- | --- | --- | --- | --- | --- |
| Numerical Evaluation Scale  (NRS) | One-dimensional scale (it measures only pain intensity and no other factors) | Numerical scale from 0 to 10, where a value of 0 indicates no pain, while a value of 10 indicates maximum pain. | 0-10 | - 1-3: mild pain, - 4 -6: moderate pain, - 7-10: severe pain | High sensitivity | Patients with limited communication skills. | NO |
| Visual Analog Scale (VAS) | One-dimensional scale (it measures only pain intensity and no other factors) | It consists of a predetermined line 100 mm long, where the left extremity corresponds to "no pain", while the right extremity corresponds to the "worst possible pain". | 0-100 mm | - 0-4 mm: no pain, - 5-44 mm: mild pain, - 45-74 mm: moderate pain, - 75-100 mm: intense pain | High sensitivity | Difficult to use in patients with visual, physical or cognitive deficits. | NO |
| Verbal Assessment Scale (VRS) | One-dimensional scale (it measures only pain intensity and no other factors) | It associates the level of pain present (No pain, Mild, Moderate, Strong, Unbearable) to a number from 0 to 4. | 0-4 | - 0= no pain - 1=mild pain - 2=moderate pain - 3= strong pain - 4= unbearable pain | Low sensitivity | Simple to use but requires language skills. | NO |
| Facies Pain Scale (FPS) | One-dimensional scale (it measures only pain intensity and no other factors) | It uses the association of six expressive mimic faces, arranged on a horizontal line, from a smiling face for “no pain” to a tearful face for “worst pain”. | 0-5 | - 0= no hurt - 1= hurts little bit - 2=hurts little more - 3=hurts even more - 4= hurts whole lot - 5= hurts worst | Low sensitivity | Patients with limited communication skills. | NO |
| Wong-Baker Pain Rating Scale (WBFPRS) | One-dimensional scale (it measures only pain intensity and no other factors) | It has a similar structure of the six-face FPS, but the score ranges from 0 to 10. | 0-10 | - 0= no hurt - 2= hurts little bit - 4=hurts little more - 6=hurts even more - 8= hurts whole lot - 10= hurts worst | Low sensitivity | Patients with limited communication skills. | NO |
| Brief Pain Inventory (BPI) | Multidimensional scale | It is structured as a qualitative and quantitative questionnaire that investigates the intensity of pain experienced in the last week and at the time of the interview and localization of pain. The BPI also provides information on the treatments performed and the relief obtained by the patient.  Furthermore, BPI evaluates the interference of pain with daily functions. | A Pain Severity Index (PSI) was derived by averaging the following pain severity items: worst and average pain and pain perceived at the time of the interview. Pain degree was defined as no pain (PSI = 0), mild pain (1 ≤ PSI ≤ 3), moderate pain (4 ≤ PSI ≤ 6), and severe pain (7 ≤ PSI ≤ 10). A Pain Interference Index (PII) was derived by averaging the interference of pain on daily functions. | - Intensity of pain is evaluated with a scale numbered 0, "no pain", to 10, "the most horrible pain imaginable." - Relief obtained with the treatments is evaluated on a scale ranging from 0% (no improvement) to 100% (improvement). - The interference of pain with daily functions is assessed by a scale numbered from 0(no interference) to 10 (total interference). | High sensitivity and specificity | Difficult to use in patients with visual, physical or cognitive deficits. | NO |
| McGill Pain Questionnaire (MPQ) | Multidimensional scale | It is used to assess both the quality and intensity of pain self-described by patients with 78 verbal pain descriptors. | 0-78 | Scores are tabulated by summing values associated with each word; scores range from 0 (no pain) to 78 (severe pain). | High sensitivity | Difficult to use in patients with visual, physical or cognitive deficits. | NO |
| Pain Assessment IN Advanced Dementia (PAINAID) | Multidimensional scale | It evaluates 5 items:  breathing, negative vocalization, facial expression, body language, consolability.  Each of these items is rated with a severity index ranging from 0 to 2 | 0-10 | - 1-3: mild pain, - 4 -6: moderate pain, - 7-10: severe pain | High sensitivity | It is designed and used for uncooperative patients with significant cognitive impairment. | NO |
| Neuropathic Pain Scale (NPS) | Questionnaire to assess the possible neuropathic component of pain | It is designed to qualify and quantify neuropathic pain and it is also useful for determining the effectiveness of different treatments. The scale includes two items that assess the global dimensions of pain intensity and pain unpleasantness and eight items that assess eight specific qualities of neuropathic pain. Each of the 10 items has a 0 to 10 numerical score. An eleventh item assesses the temporal sequence of pain as constant with intermittent increases, intermittent, or constant with fluctuation. | 0-100 | - 0: no pain - 100: worst neuropathic pain | High sensitivity and specificity | Difficult to use in patients with visual, physical or cognitive deficits. | NO |
| Leeds Assessment of Neuropathic Symptoms and Signs Pain Scale (LANSS) | Questionnaires to assess the possible neuropathic component of pain | It provides immediate clinical information and helps distinguish nociceptive pain from neuropathic pain | 0-24 | - A score of less than 12 points makes it unlikely that the patient's symptoms have neuropathic mechanisms, - while a score of 12 or higher makes it possible that the neuropathic mechanisms contribute to the pain in the patient | High sensitivity and specificity | Difficult to use in patients with visual, physical or cognitive deficits. | NO |
| Neuropathic Pain Questionnaire (NPQ) | Questionnaires to assess the possible neuropathic component of pain | It investigates the type of pain, how it changes over time, and the body sites affected. The NPQ also asks the patient to give a score from 0 to 100 for each of the 12 pain descriptors. |  | - Subjects with scores below 0 are predicted to have non-neuropathic pain, - while those with scores at or above 0 are predicted to have neuropathic pain. | High specificity and medium sensitivity | Difficult to use in patients with visual, physical or cognitive deficits. | NO |
| Pain DETECT questionnaire (PD-Q) | Questionnaires to assess the possible neuropathic component of pain | It detects components of neuropathic pain based on characteristic clinical neuropathic symptoms. It is composed of 7 items to describe pain (score 0-5), 4 pain course model (score 0-1), and 2 radiating pain (score 0-2) | 0-38 | - A score ≤ 12, indicates that pain is unlikely to have a neuropathic component, - while a score ≥ 19, suggests that pain is likely to have a neuropathic component. - A score between 13 and 18 correlates to an unclear cause for pain. | High sensitivity and specificity | It is initially developed and validated in patients with back pain, but it has shown applicability to patients with other types of neuropathic pain as well. An important advantage of this questionnaire is that it is easy for the patient to fill in, without first needing any clinical medical examination | NO |
| Douleur Neuropathique 4 DN4 | Questionnaires to assess the possible neuropathic component of pain | The questionnaire includes a series of four questions consisting of both sensory descriptors and signs related to the bedside sensory examination. Two questions (I and II) were based on the patient interview and two questions (III and IV) were based on a standardized clinical examination.  Question I have included 5 items related to the description of pain.  Question II included 4 items relating to the paresthesia/dysesthesia association within the painful area.  Question III included 4 items related to sensory deficits.  Question IV included 4 items related to evoked pain. | 0-10 | - Patients with a score ⩾ 4/10 are considered to have neuropathic pain | High sensitivity and specificity | Difficult to use in patients with visual, physical or cognitive deficits. | NO |
|  |  |  |  |  |  | Difficult to use in patients with visual, physical or cognitive deficits. |  |

Table 1 Summary Table of Pain Assessment tools
